# Non-ablative fractional laser 1940-nm treatment modulates epigenetic signatures associated with skin aging in a split-face investigation

**DOI:** 10.1038/s41598-026-56604-4

**Published:** 2026-06-30

**Authors:** Konika Patel Schallen, Kevin Schomacker, Cristiana Banila, Harry Pink, Nicolle Dest, Katherine L. R. Coleman

**Affiliations:** 1 Candela Institute for Excellence, 251 Locke Drive, 3rd Floor, Marlborough, MA 01752 USA; 2 Mitra Bio, Translation and Innovation Hub, London, UK

**Keywords:** Cell biology, Diseases, Medical research, Molecular biology

## Abstract

Skin aging is driven by multiple factors, including cumulative environmental damage and epigenetic dysregulation, yet whether energy-based therapies can durably remodel the cutaneous methylome remains unclear. We conducted a split-face, paired longitudinal study in 22 adults treated three times with a 1940-nm non-ablative fractional laser (NAFL). Epidermal samples were collected by tape-stripping from treated and contralateral control sites at baseline, after the first treatment, and after 1, 3, and 6 months. Enzymatic methyl-sequencing profiled ~ 3.8 million CpGs per sample, alongside clinical endpoints: quantitative VISIA measurements and global aesthetic scores. No significant differentially methylated regions (DMRs) were detected immediately post-treatment; however, 635 DMRs emerged 1 month after the final session, expanded through 3 months, and stabilized by 6 months. These loci were enriched for pathways related to epidermal differentiation, collagen organization, wound response, and stem-cell maintenance, with selective modulation at Polycomb-regulated and WNT-signalling genes. NAFL reversed age-associated methylation trajectories at 83.9% of CpGs showing strong differential signal in both the aging reference and NAFL-treatment. Treatment also induced transient, treatment-specific DMRs linked to immune and stress responses. Epigenetic remodelling paralleled significant clinical improvements, indicating that 1940-nm NAFL induces durable methylation changes at loci implicated in skin aging beyond transient wound-repair responses.

**Supplementary Information:**

The online version contains supplementary material available at 10.1038/s41598-026-56604-4.

## Introduction

Skin aging results from the combined effects of intrinsic biological decline and cumulative environmental exposure, leading to structural degradation, pigmentation changes, and reduced regenerative capacity^1–3^. Intrinsic aging manifests as thinning, fine wrinkles, and slower cellular turnover, while extrinsic aging, driven largely by chronic ultraviolet radiation (UVR) accelerates and intensifies these changes, producing distinct photoaging features^4,5^. UVR contributes to skin aging through cumulative lifetime exposure via distinct but overlapping mechanisms. UVA penetrates deeply into the dermis and is a major driver of oxidative stress, extracellular matrix (ECM) damage, mitochondrial dysfunction, pigmentary change, and immunomodulation^6^. By contrast, the more energetic UVB spectrum primarily affects the epidermis, where it induces direct DNA photodamage, inflammation, and mutational burden^7^.

More broadly, aging is understood to arise from the lifelong accumulation of cellular and molecular damage^8^ across multiple interconnected hallmarks, including genomic instability, telomere attrition, epigenetic alterations, loss of proteostasis, deregulated nutrient-sensing, mitochondrial dysfunction, cellular senescence, stem cell exhaustion, and altered intercellular communication^9^. Within this framework, epigenetic dysregulation represents one contributor to age-associated functional decline through changes in DNA methylation, histone modification, and chromatin organization associated with aging and disease^10,11^. Among these, DNA methylation stands out as a key biomarker, linking environmental exposures to gene expression and capturing the lasting molecular impact of factors such as UVR, pollution, nutrition, and lifestyle^12–16^.

Age-associated DNA methylation changes, including locus-specific hypermethylation at regulatory regions, mark dysregulation of pathways controlling inflammation, differentiation, tissue repair, oxidative stress, and ECM homeostasis^10,17–20^. These alterations are not fixed. Increasing evidence shows that targeted modulation of age-linked methylation states can shift molecular profiles toward those of younger tissues^21–25^. Here, consistent with prior work^25^, we define “rejuvenation” as the partial restoration of molecular or epigenetic features characteristic of youthful states, without implying full reversal of biological age or durable anti-aging effects.

Energy-based devices (EBDs) such as non-ablative fractional lasers (NAFL) are widely used for treatment of photoaging and other cutaneous aesthetic concerns through the induction of controlled microthermal zones (MTZs) that stimulate dermal remodeling while largely preserving surrounding tissue integrity. This controlled injury initiates a wound-healing cascade associated with collagen remodeling and clinical improvement in skin texture, pigmentation, and elasticity^26^. Beyond cosmetic benefit, NAFL treatment has also been associated with reduced incidence of facial keratinocyte carcinomas^27^ and actinic damage^28–31^, suggesting broader biological effects beyond structural resurfacing. Early molecular studies further indicate that EBDs modulate pathways involved in inflammation, matrix turnover, pigmentation, and regeneration^25,32–36^. However, whether these treatment-associated transcriptomic changes are underpinned by durable epigenetic remodeling, particularly at the level of DNA methylation, remains unknown.

Here, we present the first in vivo human pilot study to characterize longitudinal DNA methylation changes following treatment with a 1940-nm NAFL device. Using a split-face design and high-resolution methylation profiling, we map how NAFL modulates epigenetic signatures across pathways involved in proliferation, inflammation, pigmentation, and tissue repair. We further integrate molecular findings with clinical outcomes in pigmentation and global appearance. Comparison against a multi-cohort skin aging methylome database reveals that NAFL reverses age-associated methylation drift at the majority of responsive loci. Together, these data provide the first evidence that 1940-nm fractional laser therapy elicits remodeling of the methylome in human skin, offering insight into potential rejuvenating effects.

## Results

### Methylation profiling and study design

**Fig. 1:**
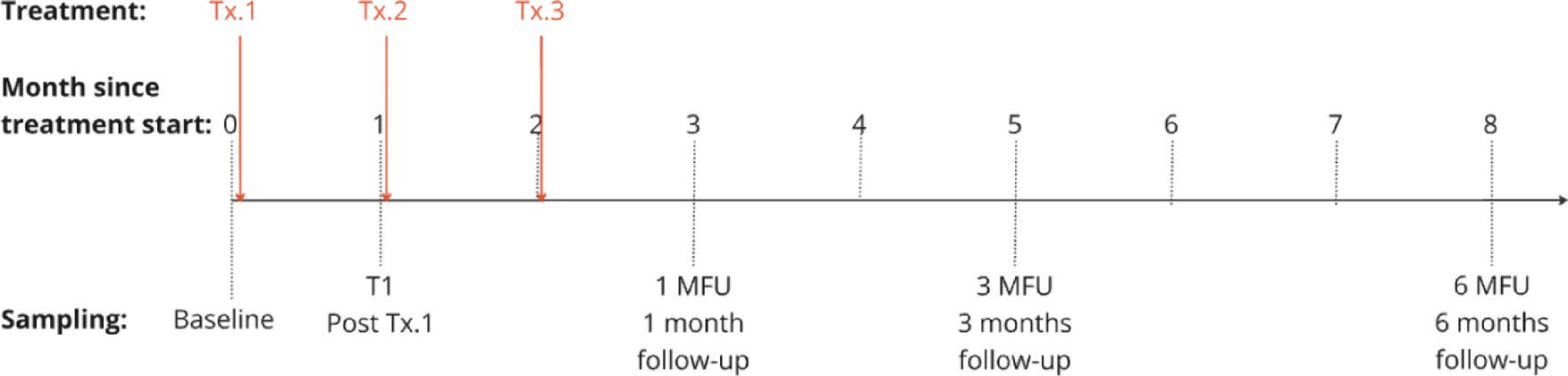
Overview of the study design and timepoints selection. An illustration and outline of the NAFL treatment strategy and sampling timepoints in the study. Abbreviations: Tx, treatment; T1, sample collection 1 after first laser treatment; Post Tx.1, post-treatment 1; MFU, month follow-up sample collection.

To map the molecular trajectory of skin rejuvenation following 1940-nm non-ablative fractional laser (NAFL), we conducted a longitudinal study enrolling a total of 22 participants. We used non-invasive tape-stripping to collect epidermal samples at Baseline, Post-Tx #1, 1-month (1MFU), 3-month (3 MFU), and 6-month (6 MFU) follow-ups (Fig. 1). The contralateral untreated site served as a within-individual control, allowing treatment-specific methylation dynamics to be isolated from inter-individual variability and environmental impacts that occur during the study duration. Genome-wide profiling was performed using enzymatic-conversion sequencing across timepoints. Cell-type deconvolution showed no significant differences across treatment nor timepoints, indicating that observed methylation differences reflect remodeling of the methylome rather than shifts in cellular makeup (Supplemental Fig. 1). This dataset constitutes the first longitudinal methylome time-course in human skin following 1940-nm NAFL treatment.

### Progressive epigenetic rejuvenation over six months

**Fig. 2:**
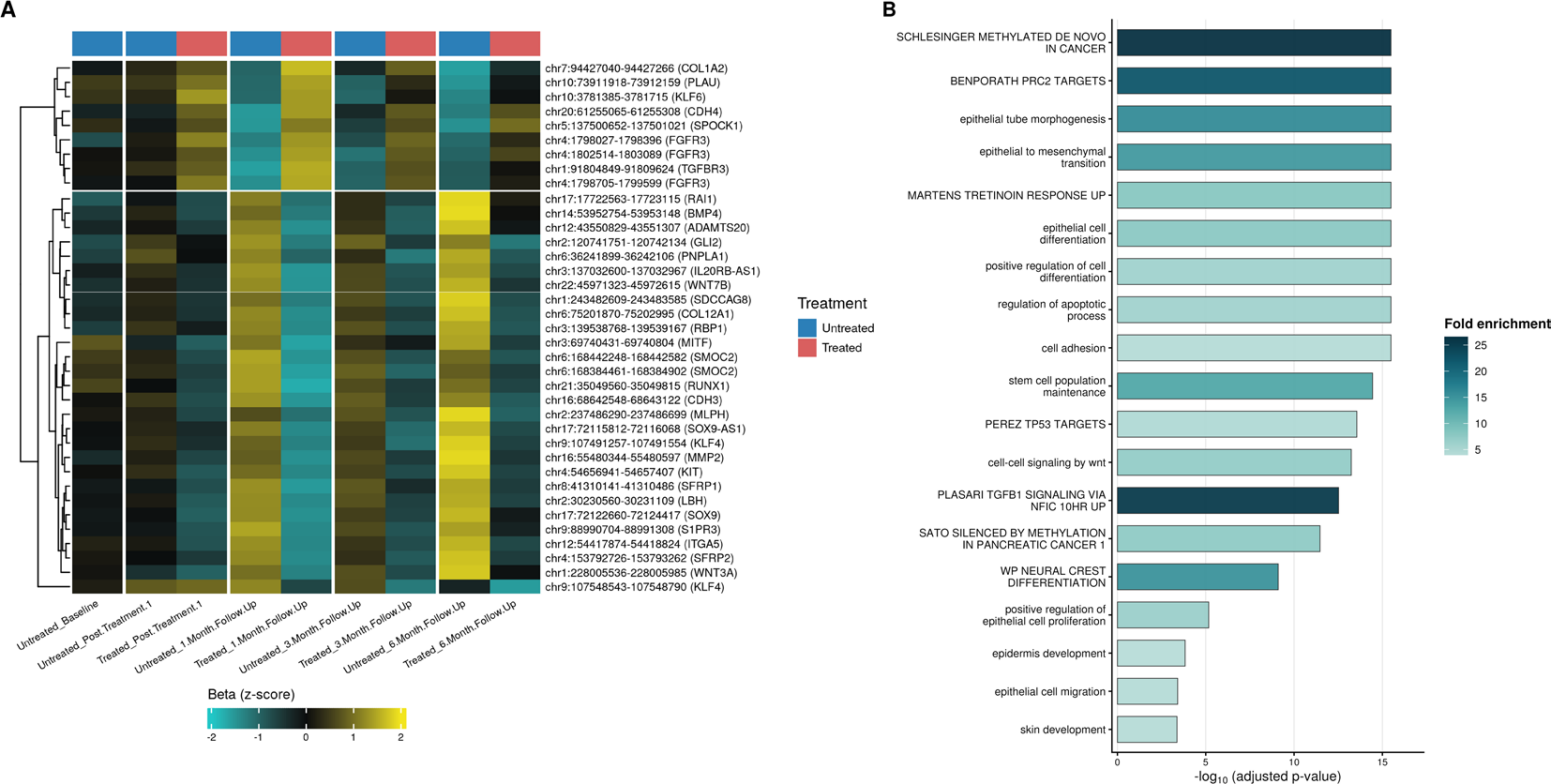
Progressive epigenetic remodeling and pathway enrichment following 1940-nm NAFL treatment. (**A**) Heatmap showing longitudinal methylation dynamics of differentially methylated regions (DMRs) in treated (red) and untreated (blue) skin. Each row represents a gene-associated region, with color indicating z-scored β-values. Directional shifts emerging at 1MFU and stabilizing by 6 MFU reflect progressive reprogramming at loci governing collagen organization (*COL1A2*), differentiation (*KLF4*), and signaling (*FGFR3*, *WNT7B*). (**B**) GO and pathway enrichment of treatment-associated DMRs ranked by adjusted *p*-value and fold enrichment. Enriched terms include epithelial differentiation, cell adhesion, stem-cell maintenance, and WNT/retinoid signaling, highlighting the transition from repair to stable molecular rejuvenation in NAFL-treated skin.

The longitudinal methylation landscape reveals that the skin’s epigenetic response to 1940-nm NAFL is progressive and coordinated (Fig. 2A–B; Table 1). A total of 635 DMRs were identified via time-series meta-analysis of the individual timepoints and met significance criteria (|Δβ| > 0.1, q < 0.05; Supplemental Table 1). The heatmap visualization revealed consistent divergence between treated and untreated skin, with progressive change at loci involved in differentiation, ECM renewal, and repair (Fig. 2A). Immediately post-treatment, methylation levels remained comparable between treated and untreated skin (Supplemental Fig. 2). Significant changes were observed at 1MFU, specific gene examples associated with skin health are highlighted Table 1. Functional enrichment revealed “epidermal differentiation”, “collagen fibril organization”, and “response to wounding” (FDR < 0.05). These changes intensified through 3MFU, indicating amplification of targeted reprogramming of the methylome as healing transitions into regenerative renewal. At 6 MFU, the pattern stabilized, suggesting the establishment of a new epigenetic steady state characterized by durable methylation reversal at genes controlling collagen synthesis, differentiation, and repair.

Functional enrichment analysis, using permutation-matched background regions and stringent filtering (FDR < 1 × 10^−3^, fold-enrichment > 2, ≥ 5 region hits), identified biological themes consistent with skin rejuvenation (Fig. 2B, Supplemental Table 2). Top GO categories included *epithelial-to-mesenchymal transition*, *epithelial differentiation*, *cell adhesion*, and *stem-cell population maintenance*, reflecting enhanced epidermal regeneration and tissue integrity. Notably, enrichment of PRC2 targets and Schlesinger de novo methylated genes in cancer indicates selective methylation resetting at Polycomb-regulated developmental loci, regions known to govern cellular plasticity during aging and rejuvenation. Pathways such as Martens retinoid response and WNT signaling further align with mechanisms of controlled keratinocyte turnover and dermal remodeling. Together, these data demonstrate that NAFL treatment elicits a stepwise change: beginning with localized modulation at regenerative genes, expanding into chromatin-level restructuring of developmental pathways, and consolidating by six months into a rejuvenated molecular architecture supporting sustained ECM integrity, epidermal renewal, and homeostatic resilience.

**Table 1:** Representative functional clusters and directionality of DMR-associated genes at 1MFU.

Functional category	Representative genes	Dominant shift	Key role
Epidermal stem cells & differentiation	*SOX9*,* KLF4*,* RUNX1*	Hypo	Stem-cell maintenance, keratinocyte differentiation, epidermal regeneration
WNT / developmental signaling	*WNT7B*,* WNT3A*,* SFRP1*,* SFRP2*,* LBH*	Hypo	Epidermal proliferation, follicular signaling, wound-repair activation
ECM organization & wound remodeling	*COL1A2*,* COL12A1*,* MMP2*,* SMOC2*,* PLAU*	Mixed (context-dependent)	Matrix remodeling, collagen organization, tissue repair dynamics
Cell adhesion & migration	*CDH3*,* CDH4*,* ITGA5*	Hypo	Keratinocyte adhesion, motility, epidermal–dermal cohesion
Growth factor & retinoid signaling	*FGFR3*,* TGFBR3*,* RAI1*,* RBP1*,* BMP4*	Predominantly Hyper	Epidermal homeostasis, regenerative vs. fibrotic signaling balance
Inflammation & repair-associated signaling	*KLF6*,* S1PR3*	Mixed	Injury response modulation, inflammatory resolution

### NAFL treatment reverses age-associated methylation patterns

**Fig. 3:**
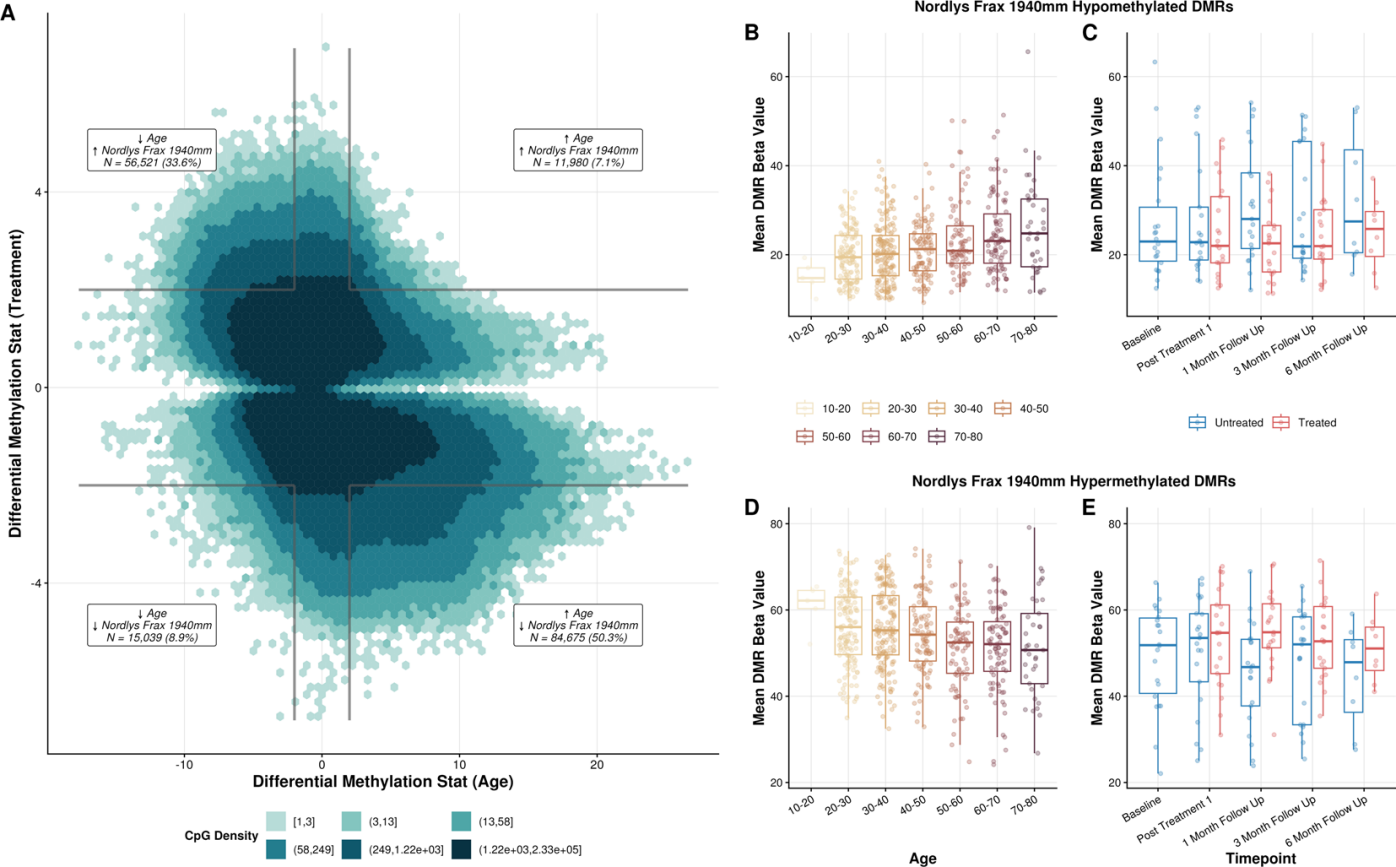
Reversal of age-associated methylation patterns following 1940-nm NAFL treatment. (**A**) Two-dimensional density (hexbin) plot showing the relationship between age-associated and treatment-associated differential methylation statistics across CpG sites. CpGs located in the upper-left and lower-right quadrants correspond to sites that gain methylation with age but lose it after NAFL, or vice versa representing rejuvenation-consistent reversal. Over 83% of CpGs changing with age and NAFL-treatment fall within these quadrants, indicating a directional correction of age-related change. (B–E) Boxplots illustrating mean β-values of hypomethylated (**B**,**C**) and hypermethylated (**D**,**E**) DMRs across age groups and treatment timepoints. Untreated samples (blue) follow the expected age-dependent trajectory, while treated sites (red) display sustained opposite-direction changes beginning at 1MFU and persisting through 6 MFU (*p* < 0.05).

To examine whether NAFL-induced methylation changes overlap with those associated with skin aging, we compared the direction and magnitude of single-CpG methylation changes across both contexts, using DSS test statistics derived independently from the treatment cohort and the MitraBio reference skin dataset^37^. The resulting two-dimensional density map (Fig. 3A) revealed a striking directional pattern, DNA methylation changes following NAFL treatment were anti-correlated with aging in human skin epidermis (Pearson’s *R*=-0.35, Spearman rho = − 0.26). Among the 168,215 CpGs showing strong differential signal in both datasets (|stat| > 2), 83.9% (141,196 CpGs) fell within the opposing quadrants, loci that hypermethylate with age yet demethylate following NAFL, and vice versa. This enrichment (Supplemental Table 3) was strongest for age-hypermethylated CpGs reversed by NAFL (3.5-fold enrichment), followed by age-hypomethylated CpGs hypermethylated by NAFL (1.6-fold enrichment). Both enrichments were statistically significant (Fisher’s-exact *p* < 2 × 10^–16^, permutation *p* < 5 × 10^− 5^, 10,000 trials). These data suggest that NAFL treatment may reverse aging-associated DNA methylation patterns. Figures 3B–E further show that, while untreated skin maintained the canonical aging trajectory, treated samples displayed a consistent anti-correlated shift beginning at 1MFU (*p* < 0.05) and persisting through 6 MFU. Together, these suggest that NAFL may reshape the methylation state by both demethylating age-hyper sites and remethylating eroded regions recapitulating rejuvenation patterns.

### Treatment-specific epigenetic changes

Beyond age-related loci, NAFL induced a subset of treatment-specific CpG changes observable via timeseries analysis in treated skin at 1MFU through 6MFU (Table 2). GO enrichment highlighted epidermal development, wound healing, extracellular matrix remodeling, and growth factor–mediated repair signaling. Early, transient responses were characterized by injury- and remodeling-associated regulators (*RUNX1*,* WNT7B*,* SFRP1*,* PLAU*,* MMP2*,* KLF6*), which stabilized over time as homeostasis- and differentiation-associated programs (*SOX9*,* KLF4*,* FGFR3*,* TGFBR3*,* PNPLA1*) became dominant. These coordinated but transient methylation changes illustrate adaptive remodeling of the epidermal methylome following controlled injury, converging on tissue repair, barrier restoration, and extracellular matrix reorganization before transitioning into a steady state.

**Table 2:** Treatment-specific differentially methylated genes reflecting transient immune, stress-response, and tissue-repair programs induced by 1940-nm NAFL.

Gene	DMR	*N* CpG	Direction	Function	GO category
*SOX9*	chr17:72122660-72124417	107	Hypo	Epidermal stem-cell maintenance	Epidermal development
*KLF4*	chr9:107491257-107491560	21	Hypo	Keratinocyte differentiation	Skin barrier formation
*RUNX1*	chr21:35049560-35049815	7	Hypo	Stem-cell activation, regeneration	Tissue regeneration
*WNT7B*	chr22:45971323-45972630	139	Hypo	Canonical WNT signaling	Developmental signaling
*SFRP1*	chr8:41310141-41310817	21	Hypo	WNT modulation, keratinocyte survival	Wound healing
*MITF*	chr3:69740431-69740804	12	Hypo	Melanocyte transcriptional control	Pigmentation
*KIT*	chr4:54656941-54657407	22	Hypo	Melanocyte survival signaling	Melanocyte development
*COL1A2*	chr7:94427040-94427266	4	Hyper	Fibrillar collagen production	ECM organization
*PLAU*	chr10:73911918-73912159	4	Hypo	Proteolytic ECM remodeling	Wound healing
*MMP2*	chr16:55480344-55480597	17	Hypo	Matrix turnover	Extracellular matrix remodeling
*ITGA5*	chr12:54417971-54418824	26	Hypo	Cell–matrix adhesion	Cell adhesion
*FGFR3*	chr4:1802514-1802998	31	Hyper	Growth factor–mediated repair signaling	Epidermal homeostasis
*TGFBR3*	chr1:91804849-91809624	9	Hyper	TGF-β signal modulation	Fibrosis vs. regeneration
*KLF6*	chr10:3781385-3781652	10	Hyper	Injury-responsive transcription	Tissue repair
*PNPLA1*	chr6:36285008-36285602	28	Hypo	Acylceramide biosynthesis	Skin barrier integrity

### Integration with rejuvenation gene sets

**Fig. 4:**
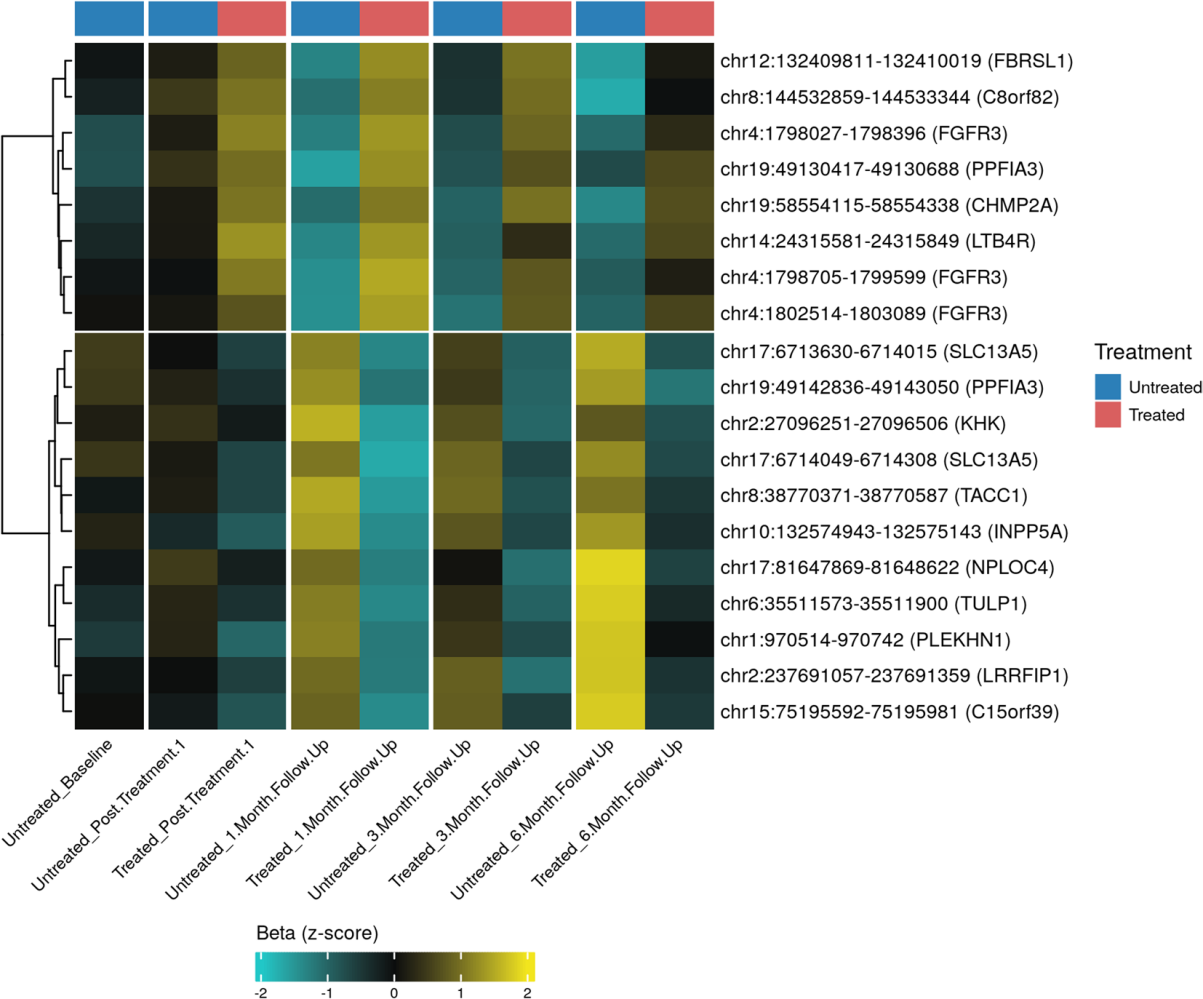
Temporal dynamics of rejuvenation-associated loci overlapping with published age-reversal gene sets. Heatmap showing longitudinal DNA methylation changes in loci overlapping rejuvenation-associated genes from Chang et al. Each row represents a DMR annotated to the nearest gene, with color indicating standardized β-values (z-scores). Treated samples (red) exhibit progressive, treatment-specific methylation reversal beginning at 1MFU, strengthening through 3 MFU and consolidating by 6 MFU, whereas untreated samples (blue) remain relatively stable or continue in the age-associated direction. Key treatment-responsive loci, including *FGFR3*,* PPFIA3*,* CHMP2A*,* SLC13A5*,* INPP5A*,* KHK*, and *PLEKHN1*, display coordinated, time-dependent methylation remodeling consistent with engagement of conserved molecular programs associated with epidermal homeostasis, structural reorganization, and metabolic rebalancing in NAFL-treated skin.

Chang et al. previously performed transcriptomic profiling of aged human skin following EBD treatment alongside untreated young controls. They define a set of “rejuvenated” genes as those whose expression shifts from aged levels toward those observed in young skin post-treatment (*n* = 1924)^25^. We identified 19 NAFL-treatment DMRs whose closest annotated gene was identified in this “rejuvenated” set (Fig. 4). The heatmap highlights time-dependent methylation reversal in key treatment-responsive genes, *including FGFR3*,* PPFIA3*,* CHMP2A*,* SLC13A5*, and *INPP5A*, that was exclusive to treated skin. These loci showed minimal change in untreated samples but displayed directional correction beginning at 1MFU, strengthening through 3 MFU, and stabilizing by 6 MFU. Functionally, the overlapping genes define a coherent network encompassing growth-factor–mediated epidermal homeostasis (*FGFR3*), extracellular-matrix and adhesion remodelling (*PPFIA3*,* CHMP2A*,* PLEKHN1*), and metabolic and signalling rebalancing (*SLC13A5*,* INPP5A*,* KHK*).

### Quantitative VISIA^®^ imaging reveals treatment-associated improvement in pigmentation and texture

**Fig. 5:**
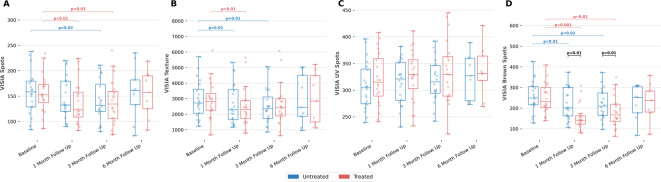
Change from baseline in four VISIA skin metrics with NAFL treatment. The four metrics: Spots, Texture, UV Spots and Brown Spots are measured at Baseline, 1-month (1MFU), 3-month (3MFU), and 6-month (6MFU) follow-up for the treated side (red) and untreated side (blue). Box plots show the median and interquartile range (whiskers extend to 1.5× IQR). For all metrics, lower values are desirable. Significance brackets denote FDR-corrected paired Wilcoxon signed-rank tests (Benjamini–Hochberg, *p* < 0.05). Black brackets indicate within-timepoint comparisons between treated and untreated sides. Red bars indicate longitudinal comparisons of the treated side versus its own baseline; blue bars indicate the same for the untreated side. Only statistically significant comparisons are shown.

Quantitative VISIA imaging demonstrated significant improvement in select pigmentation- and texture-associated parameters following 1940-nm NAFL treatment (Fig. 5). Brown spot counts showed the most robust treatment response: treated skin was significantly reduced from baseline at both 1-month (median − 38%; *p* < 0.001) and 3-month follow-up (− 21%; *p* < 0.01). Critically, brown spot reductions on the treated side were significantly greater than those on the contralateral untreated side at both 1 month (*p* < 0.01) and 3 months (*p* < 0.05), providing within-subject controlled evidence of a treatment-specific effect. Untreated skin also demonstrated smaller but significant reductions in brown spot counts at 1 and 3 months (− 14% at both timepoints; *p* < 0.01), likely reflecting seasonal or environmental influences.

Texture scores were significantly reduced from baseline on the treated side at 1 month (− 15%; *p* < 0.01), while untreated skin showed significant reductions at both 1 month (− 8%; *p* < 0.05) and 3 months (− 12%; *p* < 0.05). Spot counts were significantly reduced on the treated side at 1 month (− 9%; *p* < 0.01), and on the untreated side at 3 months (− 14%; *p* < 0.05). The treated-side spot reduction at 3 months (− 11%) was borderline significant (p_FDR = 0.053) and may reach significance in a larger cohort. No statistically significant differences were observed at the 6-month timepoint, though this subgroup was limited to 9 subjects.

## Discussion

This pilot study provides the first in vivo evidence that non-ablative fractional laser (NAFL) treatment at 1940 nm induces durable, time-dependent changes of DNA methylation in pathways relevant to skin aging. Over six months of follow-up, treated skin demonstrated progressive changes in methylation across loci regulating ECM organization, epidermal differentiation, immune signaling, developmental programs, and cellular stress responses. These results extend prior transcriptomic and histological studies of laser resurfacing^25,33,34,38^ by demonstrating that energy-based treatments can remodel the epigenetic landscape of skin, providing a potential biological mechanism underlying their sustained clinical efficacy.

A key observation of this work is the temporal separation between the immediate response to NAFL and the delayed emergence of robust DNA methylation changes. No significant DMRs were detected one month after the first treatment between treated and untreated skin as compared to baseline. In contrast to other environmental and pharmacologic exposures known to alter DNA methylation, including airborne pollutants and UV radiation, which can induce changes within hours^14,39^, demethylating drugs that act over days^40^, and smoking-related alterations that persist for decades^41^, NAFL-induced methylation changes emerged only after completion of the full treatment course of three laser applications, 28 days apart. It progressively intensified at 1- and 3-month follow-ups, stabilizing by 6 months. This delayed pattern indicates that NAFL does not directly induce rapid epigenetic change but rather initiates a program whose molecular imprint accumulates over time.

This delayed trajectory aligns with the known biological cascade of fractional photo thermolysis. NAFL induces controlled dermal microinjury, triggering an early phase marked by cytokine release and ECM degradation, followed by sustained matrix remodelling and neocollagenesis^42^. The lack of early methylation changes likely reflects this transient inflammatory response, whereas the subsequent emergence of coordinated DMRs represents the accumulating molecular signatures of treatment impact. The timing is concordant with histological evidence showing restoration of epidermal architecture and continued fibroblast activation up to six weeks after NAFL 1940 nm treatment^43^, supporting the coupling of structural regeneration with progressive epigenetic remodelling.

Similar biphasic dynamics have been reported in UVA-exposed fibroblasts, where early methylation changes localized to active defense genes and later hypermethylation occurs at silenced developmental loci^44^. Our findings are consistent with this model: acute injury does not immediately translate into methylome changes, but regenerative signaling over weeks to months leaves a durable molecular signature. Given that DNA methylation marks can persist through cell divisions^45^, such remodeling may underpin the long-term clinical benefits observed after treatment.

Many remodelled loci overlapped regions associated with age-related epigenetic drift, the progressive dysregulation of DNA methylation observed in aging skin^46^. This process is characterized in part by gradual hypermethylation of Polycomb Group Target (PCGT) genes developmental regulators that are bivalently marked in embryonic stem cells and critical for maintaining differentiation potential^47^. Consistently, NAFL-responsive DMRs were enriched near Polycomb Repressive Complex 2 (PRC2) target loci, a subset of these genes known to undergo age-associated hypermethylation^48^. Together, these findings suggest that NAFL may remodel methylation at regulatory regions implicated in established epigenetic drift.

Bioinformatic analyses have further identified age-associated methylation “hotspots” across multiple tissues that converge on stem-cell differentiation and WNT-signaling pathways^49^, an axis central to keratinocyte proliferation, fibroblast activation, pigmentation, wound repair, and hair follicle stem cell maintenance^50–52^. In our study, NAFL-responsive DMRs were enriched in key components of this pathway (*SFRP1*,* SFRP2*,* WNT3A*,* WNT7B*, and *LBH*) with directional methylation changes opposing known age-related drift. These findings indicate that fractional photo thermolysis modulates methylation at WNT-responsive and developmental loci previously implicated in age-associated epigenetic remodeling, consistent with engagement of regulatory pathways involved in epidermal renewal and tissue repair. This interpretation is aligned with prior work demonstrating that controlled photothermal stress activates mitochondrial and redox-mediated repair mechanisms^53^ and enhances stem-cell proliferation in vitro^54^.

Importantly, hundreds of DMRs in this study shifted in the opposite direction to age-related drift within ECM, immune, and differentiation pathways, supporting the concept that effective methylation remodeling may occur through targeted modulation of age-sensitive regulatory hubs rather than through broad resetting of the epigenome^14,18,55^.

Concurrent methylation remodeling was observed at FN1 and TGFBR3, loci involved in ECM organization and TGF-β signaling pathways associated with fibrotic and reparative tissue responses. Given that fibronectin overexpression and sustained TGF-β signaling have been implicated in hypertrophic scarring and age-related tissue rigidity^56,57^, modulation of these loci may reflect remodeling of epigenetic regulatory regions relevant to wound-healing programs. This interpretation is consistent with prior histologic studies demonstrating organized dermal remodeling without fibrosis following NAFL treatment^58^.

Epigenetic changes were also detected in genes associated with keratinocyte differentiation, stress responses, and proliferative regulation. These included FGFR3, a receptor tyrosine kinase implicated in epithelial proliferation, tissue repair, and the pathogenesis of benign and malignant skin lesions^59–61^, and UBE2I (UBC9), the sole E2 conjugating enzyme of the SUMOylation pathway, which contributes to differentiation, DNA damage responses, and genomic maintenance^62^. Dysregulation of these pathways has been associated with fibrosis, pigmentary disorders, and carcinogenesis^63–65^.

Additional methylation change was observed at loci implicated in epidermal proliferation and regenerative regulation, including HOXB4, a transcription factor involved in epithelial stem-cell self-renewal whose promoter hypermethylation has been associated with poor prognosis in epithelial cancers^66^ and whose expression in skin localizes to proliferative layers of malignant and hyperproliferative lesions^67^. Similar changes were also observed in PPP1R18 and PPP1R26, phosphatase regulators implicated in keratinocyte proliferation and metastatic behavior^68^.

We further observed NAFL-associated methylation changes at genes involved in immune and stress-response pathways (*e.g. LILRB4*,* S1PR3*,* RORA*,* ALOX15*). The NF-κB signaling network sits at the intersection of these processes, maintaining inflammatory tone and enforcing senescence-associated gene expression. Prior work by Adler et al.^69^ demonstrated that aged murine tissues exhibit persistent NF-κB, activation accompanied by elevated expression of inflammatory mediators and cell-cycle inhibitors, while inhibition of NF-κB reduced markers of senescence, restored epidermal thickness, and enhanced proliferative capacity while maintaining normal differentiation and tissue organization. These findings suggest that the aged phenotype is not merely degenerative but is actively sustained through NF-κB activity, highlighting this pathway as a promising target for age-associated epigenetic remodeling. Supporting this concept, prior transcriptomic analysis showed that genes associated with a rejuvenated skin state were significantly enriched for NF-κB targets^25^. Collectively, these studies position NF-κB as a key mediator of age-associated inflammatory signaling and a potentially reversible pathway responsive to energy-based interventions.

The molecular remodeling observed in treated skin corresponded with visible clinical improvement in pigmentation, texture, and overall appearance. Peak clinical benefit was observed at the 1-month follow-up, as evidenced by maximal improvement in quantitative VISIA pigmentation metrics, global aesthetic ratings, and subject satisfaction scores. Although many clinical endpoints remained improved relative to baseline at later timepoints, the magnitude of benefit attenuated over time and did not retain statistical significance at 6 months. Interpretation of the 6-month findings should be made cautiously, however, given the reduced sample size at this optional follow-up visit (*n* = 10). This temporal pattern is broadly consistent with prior studies of 1927-nm fractional thulium lasers, in which peak clinical improvement was observed within 1–3 months after treatment, followed by sustained but attenuated responses at later follow-up^70,71^.

The parallel improvement in clinical and molecular endpoints suggests that visible tissue remodeling following NAFL treatment is accompanied by measurable epigenetic changes within pathways relevant to ECM organization, epidermal differentiation, pigmentation, and inflammatory regulation. By 6 months, the epidermal methylome stabilized into a remodeled configuration characterized by methylation changes opposing known age-associated drift at multiple loci, supporting the interpretation that 1940-nm NAFL induces durable molecular change beyond the acute wound-healing response. These findings highlight the potential utility of epigenetic biomarkers as objective tools for assessing biological response to energy-based aesthetic interventions.

Several limitations warrant consideration. The modest cohort size, particularly at the six-month time point, limits statistical power and generalizability. The cohort size was too limited for robust stratified analyses by phototype, age, or sex while accounting for repeated measures at the individual level, which was prioritized in the DMR analysis. Larger studies will be required to assess these effects. Moreover, the absence of corresponding transcriptomic or proteomic validation precludes definitive conclusions about downstream functional consequences. Future investigations should integrate multi-omics profiling to link methylation dynamics with gene expression, protein activity, and clinical endpoints over extended follow-up periods. Expanding analyses across diverse skin types, anatomical sites, and age groups, along with more even gender distribution will be essential to capture biological variability and confirm reproducibility.

Collectively, these findings position 1940-nm non-ablative fractional laser therapy as more than a structural resurfacing tool, demonstrating that its clinical effects are accompanied by coordinated epigenetic change across loci involved in differentiation, extracellular matrix organization, inflammation, and cellular stress responses. The observed overlap with canonical age-associated methylation drift suggests that NAFL engages epigenetic regulatory regions implicated in skin aging biology. By linking a clinically established rejuvenation modality with quantifiable molecular remodeling, this pilot provides a framework for understanding the durable biological effects of fractional photo thermolysis and supports further exploration of epigenetic biomarkers in the development and evaluation of precision aesthetic therapies.

## Methods

### Participant selection

Twenty-four subjects with Fitzpatrick Skin Types II–IV and ages ranging from 20 to 64 years were enrolled. Two participants were excluded due to clinical study protocol non-compliance: one prior to treatment and one after the first session, resulting in 22 subjects completing the full three-session protocol and 3-month follow-up. An optional 6-month visit was completed by 10 of the 22 participants.

Key exclusion criteria included any medical condition potentially affecting treatment or evaluation, use of topical antibiotics or retinoids within 30 days, skin resurfacing in the treatment area within 3 months (or CO₂ laser within 6 months), or prior aesthetic procedures (e.g., dermal fillers, neuromodulators) in the treatment area within 3 months.

All participants provided written informed consent, including consent for clinical photography.

All volunteer data has been collected in accordance with ethics and regulations. Volunteer recruitment was performed at one site in the US. The study was approved by Allendale Investigational Review Board (AIRB), registered with OHRP and FDA, under FRX23001. The study was conducted in accordance with the ethical principles of the Declaration of Helsinki. All participants provided written informed consent prior to inclusion. Electronic data was anonymized and stored by Candela.

### Laser treatment

Subjects received topical non-ablative fractional laser (NAFL, 1940 nm) treatment on one side of the face (left or right), with the contralateral side serving as an untreated control. Topical anesthetic was applied under occlusion for 30 min, followed by antiseptic cleansing and skin marking. Each subject received a total of three treatments, 28 days apart.

Treatment was delivered using the 1940-nm handpiece equipped with SoftCool™ cooling, which provides a directed stream of cool air for enhanced comfort. Laser energy ranged from 12.2 to 20 mJ with pulse durations averaging 5.39 ms, and treatment density varied between 30 and 60% overlap. Penetration depths ranged from 170 to 270 μm. Each session included 2–6 passes using both vertical and horizontal orientations to ensure even coverage. The average energy delivered per treatment was 0.43 kJ. Control sites underwent identical preparation without laser activation. Treatments were administered in three sessions at 4-week (± 1 week) intervals. All subjects were advised to avoid sun exposure and use broad-spectrum sunscreen (SPF ≥ 30) throughout the study.

### Skin tape-stripping

Non-invasive skin sampling was performed via tape stripping at baseline, 4 weeks post-treatment #1, 1 month and 3 months post-final treatment, and optionally at 6 months from both treated and untreated sites of the face. Following cleansing with 70% isopropanol, the forehead sampling site was marked using a sticker frame for consistency and tape disks were repeatedly applied and removed. Eight disks per sample were collected into 10mL containers with 2 mL DNA stabilizing solution and stored at 4 °C until shipment at ambient temperature.

### Sample processing for epigenetic sequencing

Genomic DNA was isolated from tape strips using a modified semi-automated version of the Quick-DNA HMW MagBead Kit protocol (ZymoResearch, Irvine, CA, USA) as previously described in Banila et al.^72^. Libraries were prepared using a modified semi-automated version of NEBNext^®^ Enzymatic Methyl-seq (EMseq) (New England Biolabs, Ipswich, MA, USA) as per manufacturer’s instructions. Libraries were pooled equimolarly and subjected to hybridization-based target enrichment using Twist Bioscience Human methylome panel (Twist Bioscience, San Francisco, CA, USA) and were quality controlled on a TapeStation 4150 HSD1000 (Agilent, Santa Clara, CA, USA). The process was performed on174 skin samples collected at baseline and follow-up points. 172 samples passed quality control checks and showed optimal concentrations ranging from 0.1 ng/µL to 2.76 ng/µL in volumes of 200 µl (excluding outliers). 2 samples had concentrations below 0.05 ng/µL and were outside of the quantification range and therefore excluded.

Sequencing was performed on Illumina NovaSeq X flow cells (Illumina, San Diego, CA, USA). High-quality sequencing data were successfully generated from 171 out of 172 samples. The reads were processed through an internal bioinformatics pipeline, as previously described in Menendez Vazquez et al.^37^ Briefly reads are trimmed using TrimGalore (v0.6.6), reads are aligned to GRCh38.p14 with bwa-meth (v0.2.7), the resulting BAMs are sorted and have duplicates removed using samtools (v1.20). Methylation status is quantified at CG positions using methyldakel extract (v0.6.1) with the optional --minConversionEfficiency 0.9 argument specified. methyldakel extract is also performed at CHH/CHG loci to calculate EM-seq conversion efficiency.

To ensure robust and interpretable comparisons, stringent filtering criteria were applied. CpG sites located on chromosomes X, Y, and mitochondrial DNA were excluded. Samples with mean coverage below 15X or conversion efficiency below 99% were removed, resulting in 167 high-quality samples out of 171. CpGs exhibiting no variance across these samples, or mean coverage under 8X were also excluded. After filtering, methylation values were retained for 3.82 million CpGs out of the 3.99 million sites included in the custom panel. All 167 of 171 samples fell within expected beta value distribution ranges. Cell-type deconvolution was performed with EpiDISH R package using Muse et al. (2022) skin-reference package.

### Differential methylation

Differential methylation analysis was performed using a beta-binomial model with DSS-smooth^73^. We performed differentially methylated cytosine (DMC) analysis for each timepoint individually (treated-untreated) using a paired design controlled for participant effects. In addition, we performed time-series meta-analysis using Stouffer’s signed method, combined Z-scores were calculated across timepoints; Z_combined = (Σ Z_j) / √k, where Z_j = sign(t_j) × Φ⁻¹(1 - p_j/2) for timepoint j, and k is the number of timepoints. Combined p-values were obtained as: p_combined = 2 × Φ(-|Z_combined|). Combined p-values were adjusted for multiple-testing using the Benjamini-Hochberg method. Differentially methylated regions (DMRs) were called using the callDMR method in DSS using the following thresholds; p.threshold = 1e-4, pct.sig = 0.5, minlen = 200, dis.merge = 2000.

Functional enrichment of DMR regions was carried out using rGREAT^74^, using Gene Ontology (GO) terms from the Biological Process (BP) and Molecular Function (MF) ontologies as well the MSigDB curated gene sets (C2) the background set was made up of 2000 random regions per DMR matched for size and chromosome distribution. Thresholds for significant enrichment were set at p-adjust <1e-3, fold-enrichment > 2, region-hits > 5.

### Age-associated CpGs

To identify age-associated CpGs in human skin epidermis, we utilised internal Mitra Bio samples previously used for epigenetic clock training^37^(n = 685, aged 18–90 years, median = 43). These samples were collected across 10 unique cohorts. Samples were collected and processed identically to those described above. Differential methylation analysis was performed using the formula “~Age + Cohort” with DMLfit.multiFactor and DMLtest.multiFactor from the DSS R package. The resulting single-CpG test statistic provides a continuous, directional measure of age association, positive values indicating hypermethylation with advancing age and negative values hypomethylation, enabling genome-wide comparison of age trajectories with treatment-induced methylation changes without the need for binary thresholding.

CpGs were stratified into four quadrants by the sign of their age and treatment test statistics (each filtered at |stat| > 2): age-hyper/treatment-hyper, age-hyper/treatment-hypo, age-hypo/treatment-hyper, and age-hypo/treatment-hypo., Enrichment for each quadrant was assessed using a fisher’s exact test. These results were validated with empirical permutation tests by randomly shuffling treatment-significance labels across CpGs with fixed age-significance, and recalculating overlaps.

### Clinical assessments

Subjects were evaluated at baseline and longitudinal follow-ups. VISIA measurements were collected from *n* = 20/22. Two subjects were excluded as they received laser treatments on the forehead only and VISIA performs full-face analysis. Standardized facial images (frontal, 45°, and lateral profiles) were acquired using the VISIA^®^ imaging system (Canfield Scientific, Fairfield, NJ). Quantitative VISIA^®^ analysis included absolute feature counts for spots, brown spots, UV spots, and texture. Pigmentary lesions assessed comprised dyschromic skin lesions including freckles, acne scars, and dyspigmentation (“spots”); lesions associated with photodamage (“UV spots”); and lentigines and melasma (“brown spots”). Texture analysis quantified raised and depressed skin surface variations contributing to overall skin smoothness.

## Supplementary Information

Below is the link to the electronic supplementary material.

Supplementary Material 1

Supplementary Material 2

## Data Availability

‘The datasets generated during and/or analysed during the current study are available in the Gene Expression Omnibus (GEO) repository, under [GSE315794](https:/www.ncbi.nlm.nih.gov/geo/query/acc.cgi? acc=GSE315794) to be found at [https://www.ncbi.nlm.nih.gov/geo/query/acc.cgi? acc=GSE315794](https:/www.ncbi.nlm.nih.gov/geo/query/acc.cgi? acc=GSE315794) For review access please use code qdefwmamljebzol.’
