# Supplementary material for: Non-ablative fractional laser 1940-nm treatment modulates epigenetic signatures associated with skin aging in a split-face investigation
Source: Sci Rep. 2026 Jun 30;16:17695. doi: 10.1038/s41598-026-56604-4 (PMC13319740; doi:10.1038/s41598-026-56604-4)
Supplement: Supplementary file 1 — Supplementary Material 1 [file 41598_2026_56604_MOESM1_ESM.pdf]

Supplementary Figures and Tables

Non-ablative fractional laser 1940-nm treatment modulates epigenetic signatures associated with skin aging in a split-face investigation

Konika Patel Schallen, MD[1]; Kevin Schomacker, PhD[1]; Cristiana Banila, PhD[2]; Harry Pink, PhD[2]; Nicolle Dest, MSN, RN[1]; Katherine LR Coleman, MS[1]

[1]Candela Institute for Excellence, Marlborough, MA

[2]Mitra Bio, Translation and Innovation Hub, London, UK

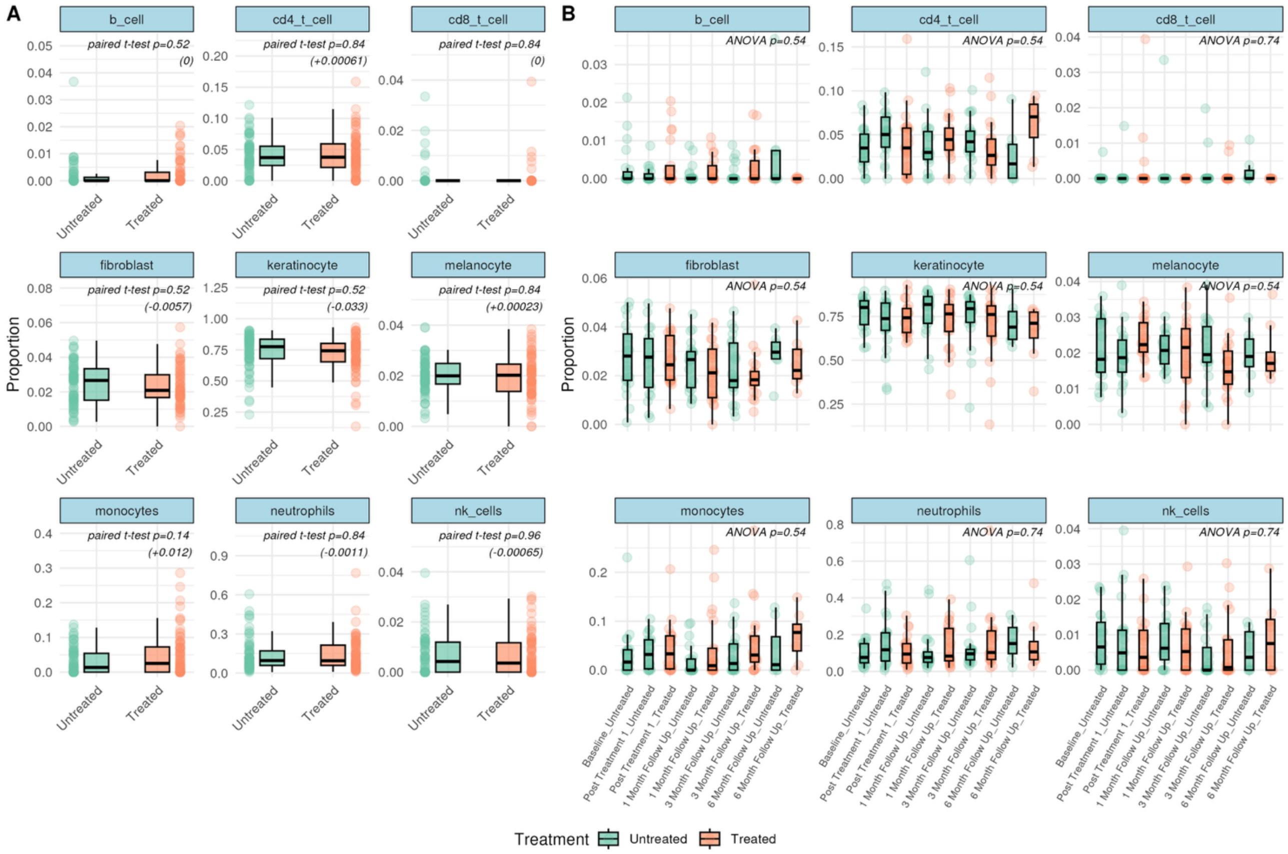

Supplemental Figure 1: Cell-type composition across treatment conditions and timepoints. (**A**) Estimated proportions of major skin and immune cell populations in paired untreated and treated samples. Boxplots show distributions for B cells, CD4^{+} and CD8^{+} T cells, fibroblasts, keratinocytes, melanocytes, monocytes, neutrophils, and NK cells, with individual samples overlaid. Paired tests were used to assess differences, with p-values indicated. (**B**) Longitudinal analysis of cell-type proportions across the treatment course. Boxplots display distributions at each timepoint for untreated (green) and treated (orange) samples; significance was assessed by ANOVA.

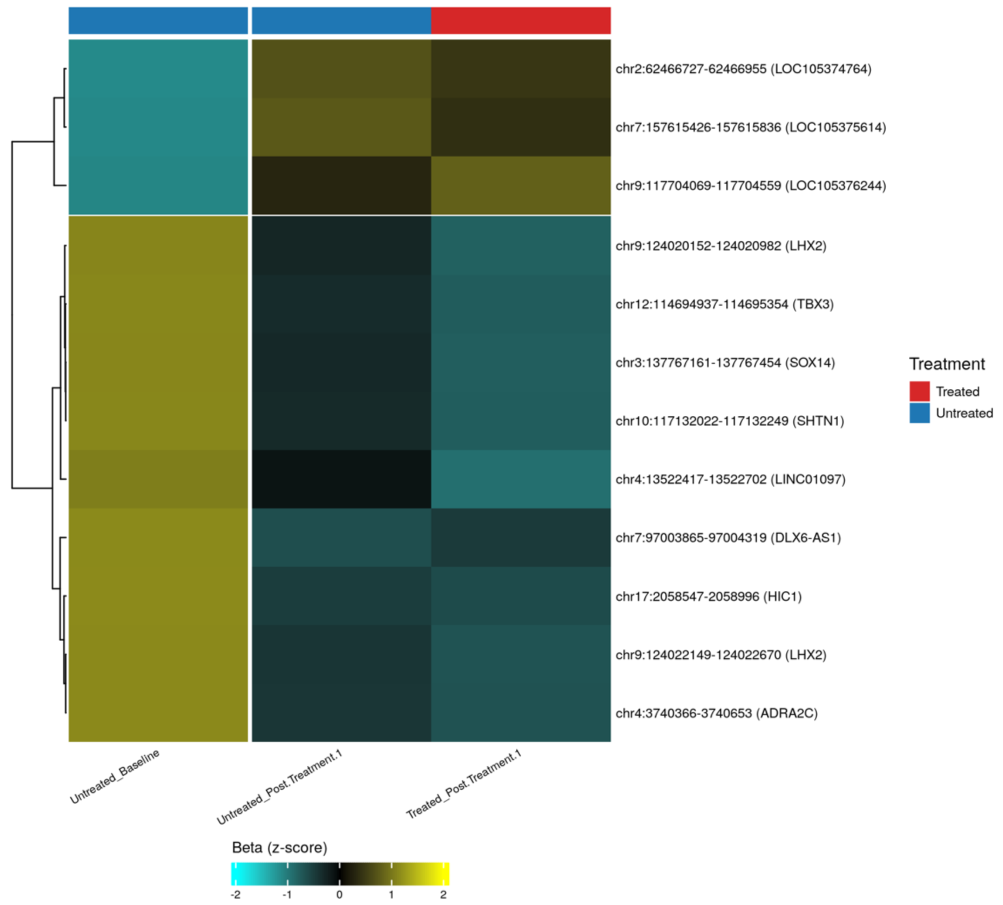

Supplemental Figure 2: Differential methylation patterns post-treatment 1, untreated vs treated as compared to baseline. Heatmap of selected differentially methylated regions (DMRs) showing standardized effect sizes (beta values, z-scored) across untreated baseline, untreated post-treatment 1, and treated post-treatment 1 samples. Each row represents a genomic locus annotated with the nearest gene, and each column represents a sample group. Hierarchical clustering was applied to group loci with similar methylation patterns. Colour scale indicates relative methylation levels (yellow, higher; blue, lower). Distinct clusters highlight loci exhibiting treatment-associated shifts, with several regions showing increased methylation in treated post-treatment samples compared to untreated conditions. Although there are differences between baseline, there are no significant differences observed between untreated post-treatment 1 and treated post-treatment 1.

Supplemental table 1: Details of the resulting 635 differentially methylated regions (DMRs) from Timeseries analysis

chr	start	end	length	nCG	areaStat	SYMBOL	GENENAME	distanceToTSS	annotation
**chr20**	45097668	45098539	872	111	-473.6819663170800	KCNS1	potassium voltage-gated channel modifier subfamily S member 1	2573	Promoter (2-3kb)
**chr9**	124012553	124014131	1579	101	-429.58816516138400	LHX2	LIM homeobox 2	196	Promoter (<=1kb)
**chr17**	72122660	72124417	1758	107	-425.50250069216800	SOX9	SRY-box transcription factor 9	1640	Promoter (1-2kb)
**chr22**	45971323	45972630	1308	139	-423.2846591787940	WNT7B	Wnt family member 7B	85	Promoter (<=1kb)
**chr13**	24745630	24746805	1176	102	-392.23343490739600	RNF17	ring finger protein 17	-853	Promoter (<=1kb)
**chr15**	37096228	37098178	1951	91	-354.25987635355700	MEIS2	Meis homeobox 2	0	Promoter (<=1kb)
**chr2**	218781546	218782688	1143	86	-344.0639329124820	CYP27A1	cytochrome P450 family 27 subfamily A member 1	0	Promoter (<=1kb)
**chr14**	36582974	36584524	1551	85	-337.3961779014650	NKX2-8	NK2 homeobox 8	-360	Promoter (<=1kb)
**chr15**	76337294	76338432	1139	98	-328.9322990518530	ISL2	ISL LIM homeobox 2	0	Promoter (<=1kb)
**chr8**	22690027	22690914	888	82	-320.32710040847500	LOC124901908	uncharacterized LOC124901908	0	Promoter (<=1kb)
**chr7**	64888529	64889772	1244	87	-314.1345882290190	LOC441239	uncharacterized LOC441239	328	Promoter (<=1kb)
**chr5**	139847926	139848693	768	100	-314.06278971663000	NRG2	neuregulin 2	23110	Exon (ENST00000504413.1/ENST00000504413.1, exon 1 of 3)
**chr13**	50132100	50133626	1527	81	-288.11712777149900	DLEU2	deleted in lymphocytic leukemia 2	-6380	Intron (ENST00000486895.5/10301, intron 1 of 1)
**chr6**	136923302	136924027	726	66	-284.4541372171170	SLC35D3	solute carrier family 35 member D3	1001	Promoter (1-2kb)
**chr16**	56635034	56635866	833	66	-282.1943168429850	MT1JP	metallothionein 1J, pseudogene	0	Promoter (<=1kb)
**chr17**	7383690	7384571	882	74	-274.56817421386800	TNK1	tyrosine kinase non receptor 1	0	Promoter (<=1kb)
**chr6**	170296784	170297633	850	67	-271.28956167183400	FAM120B	family with sequence similarity 120 member B	1463	Promoter (1-2kb)

**chr5**	3596504	3597595	1092	74	-269.91726688277300	IRX1	iroquois homeobox 1	672	Promoter (<=1kb)
**chr19**	36605108	36605813	706	69	-259.455404673107	ZNF529	zinc finger protein 529	0	Promoter (<=1kb)
**chr1**	243482609	243483585	977	72	-253.95939547790500	SDCCAG8	SHH signaling and ciliogenesis regulator SDCCAG8	-4230	Intron (ENST00000366541.8/10806, intron 16 of 17)
**chr10**	101236198	101237588	1391	77	-249.17285920364200	LBX1-AS1	LBX1 antisense RNA 1	130	Promoter (<=1kb)
**chr20**	45316482	45317095	614	57	-246.94137684366200	RBPJL	recombination signal binding protein for immunoglobulin kappa J region like	557	Promoter (<=1kb)
**chr1**	200873446	200874182	737	68	-242.4460556148700	GPR25	G protein-coupled receptor 25	465	Promoter (<=1kb)
**chr6**	163630432	163631290	859	60	-239.2535997734850	LOC124901499	uncharacterized LOC124901499	-40287	Distal Intergenic
**chr6**	41638484	41639060	577	56	-233.53119336462500	MDFI	MyoD family inhibitor	0	Promoter (<=1kb)
**chr14**	36522924	36524470	1547	62	-232.9248363353090	NKX2-1	NK2 homeobox 1	-1775	Promoter (1-2kb)
**chr14**	36579817	36580763	947	57	-229.27996937299800	NKX2-8	NK2 homeobox 8	1851	Promoter (1-2kb)
**chr11**	62926022	62926712	691	60	-228.61497615825000	CHRM1	cholinergic receptor muscarinic 1	-4215	Distal Intergenic
**chr10**	100519269	100520034	766	74	-227.22208884589100	SEC31B	SEC31 homolog B, COPII coat complex component	0	Promoter (<=1kb)
**chr17**	77373402	77374220	819	65	-219.24464597664200	SEPTIN9	septin 9	0	Promoter (<=1kb)
**chr1**	170660212	170661168	957	51	-218.25438419421300	PRRX1	paired related homeobox 1	-1560	Promoter (1-2kb)
**chr5**	93588057	93588621	565	66	-216.73789177788200	NR2F1	nuclear receptor subfamily 2 group F member 1	597	Promoter (<=1kb)
**chr7**	155461950	155465746	3797	57	-212.18132391399900	EN2	engrailed homeobox 2	3821	3' UTR
**chr17**	81647825	81648640	816	62	-207.22854189559500	NPLOC4	NPL4 homolog, ubiquitin recognition factor	0	Promoter (<=1kb)
**chr12**	114671521	114672337	817	60	-206.0944956809940	TBX3	T-box transcription factor 3	7596	3' UTR
**chr8**	37697821	37698395	575	56	-204.45119699140300	ZNF703	zinc finger protein 703	2039	Promoter (2-3kb)

**chr7**	114082749	114083675	927	53	-201.17848331173200	FOXP2	forkhead box P2	-2652	Promoter (2-3kb)
**chr7**	128032420	128033051	632	62	-200.711303140266	LRRC4	leucine rich repeat containing 4	-313	Promoter (<=1kb)
**chr9**	136821297	136821763	467	61	-197.05368589478100	RABL6	RAB, member RAS oncogene family like 6	0	Promoter (<=1kb)
**chr2**	25276913	25277580	668	47	-196.00640082966900	DNMT3A	DNA methyltransferase 3 alpha	-1393	Promoter (1-2kb)
**chr9**	124016591	124017604	1014	61	-195.94506951728200	LHX2	LIM homeobox 2	1194	Promoter (1-2kb)
**chr17**	17722541	17723204	664	55	-194.94153878960800	RAI1	retinoic acid induced 1	40046	Intron (ENST00000353383.6/10743, intron 1 of 5)
**chr12**	48997071	48997936	866	58	-190.8390765257310	DDN-AS1	DDN and PRKAG1 antisense RNA 1	-431	Promoter (<=1kb)
**chr9**	88990630	88991299	670	45	-187.8468242364330	S1PR3	sphingosine-1-phosphate receptor 3	0	Promoter (<=1kb)
**chr17**	61454469	61455033	565	59	-185.68302246920700	TBX4	T-box transcription factor 4	-1413	Promoter (1-2kb)
**chr4**	54092804	54094324	1521	48	-185.16227298434200	GSX2	GS homeobox 2	-5199	Intron (ENST00000507166.5/5156, intron 12 of 23)
**chr20**	44115992	44116382	391	50	-183.63078967356500	TOX2	TOX high mobility group box family member 2	61641	Exon (ENST00000372980.4/57158, exon 4 of 6)
**chr16**	66579043	66579621	579	54	-183.6067117122630	CMTM2	CKLF like MARVEL transmembrane domain containing 2	0	Promoter (<=1kb)
**chr2**	156329061	156329803	743	59	-181.58923377996400	NR4A2	nuclear receptor subfamily 4 group A member 2	0	Promoter (<=1kb)
**chr1**	35577343	35577874	532	47	-180.11332655450800	TFAP2E-AS1	TFAP2E antisense RNA 1	0	Promoter (<=1kb)
**chr16**	68642548	68643122	575	46	-180.03650053406100	CDH3	cadherin 3	-1871	Promoter (1-2kb)
**chr17**	61405103	61405613	511	60	-179.10372067334700	TBX2	T-box transcription factor 2	4738	3' UTR
**chr10**	103692408	103693013	606	45	-178.90641502391800	SH3PXD2A	SH3 and PX domains 2A	610	Promoter (<=1kb)
**chr19**	45217309	45217894	586	45	-177.70592960211900	EXOC3L2	exocyst complex component 3 like 2	27513	Exon (ENST00000413988.3/90332, exon 10 of 12)

**chr14**	101705606	101706108	503	51	-177.6296413285140	LINC00239	long intergenic non-protein coding RNA 239	-24329	Intron (ENST00000557778.1/105370676, intron 1 of 2)
**chr1**	26224948	26225861	914	55	-174.36947484434	ZPLD2P	zona pellucida like domain containing 2, pseudogene	0	Promoter (<=1kb)
**chr5**	135027747	135028379	633	55	-173.75752136898300	PITX1	paired like homeodomain 1	3631	3' UTR
**chr1**	12595144	12596362	1219	49	-173.03966832220900	DHRS3	dehydrogenase/reductase 3	0	Promoter (<=1kb)
**chr14**	73712150	73713534	1385	44	-171.179608621376	PNMA1	PNMA family member 1	850	Promoter (<=1kb)
**chr2**	130963117	130963609	493	52	-169.0869128849750	ARHGEF4	Rho guanine nucleotide exchange factor 4	-33	Promoter (<=1kb)
**chr16**	54283479	54284402	924	45	-167.37323041763100	IRX3	iroquois homeobox 3	1453	Promoter (1-2kb)
**chr17**	39165230	39165641	412	37	-167.03638754396500	ARL5C	ADP ribosylation factor like GTPase 5C	520	Promoter (<=1kb)
**chr1**	94927401	94927905	505	36	-164.6966472545520	CNN3-DT	CNN3 divergent transcript	0	Promoter (<=1kb)
**chr12**	43550829	43551351	523	37	-164.2903759627070	ADAMTS20	ADAM metallopeptidase with thrombospondin type 1 motif 20	570	Promoter (<=1kb)
**chr15**	52791041	52791695	655	49	-163.97032468774800	ONECUT1	one cut homeobox 1	0	Promoter (<=1kb)
**chr20**	40692456	40693006	551	37	-163.18454210299700	MAFB	MAF bZIP transcription factor B	-3220	Distal Intergenic
**chr14**	92514365	92514850	486	46	-162.41814314103700	LOC124903365	uncharacterized LOC124903365	561	Promoter (<=1kb)
**chr22**	31344413	31345317	905	44	-162.19956933698400	PATZ1	POZ/BTB and AT hook containing zinc finger 1	459	Promoter (<=1kb)
**chr18**	79399105	79399569	465	29	-161.47427654231500	NFATC1	nuclear factor of activated T cells 1	-705	Promoter (<=1kb)
**chr17**	18382831	18383396	566	46	-156.63925362261800	EVPLL	envoplakin like	-3008	Exon (ENST00000399134.5/645027, exon 6 of 11)
**chr10**	42933530	42934313	784	43	-153.286878348541	LINC01264	long intergenic non-protein coding RNA 1264	47194	Distal Intergenic
**chr8**	53878892	53879663	772	47	-152.87327448410500	RGS20	regulator of G protein signaling 20	-221	Promoter (<=1kb)

**chr2**	70904560	70905362	803	43	-152.63207559087500	ATP6V1B1	ATPase H+ transporting V1 subunit B1	3762	Intron (ENST00000234392.3/25806, intron 1 of 2)
**chr5**	42991879	42992411	533	43	-151.9806319743170	FLJ32255	uncharacterized LOC643977	413	Promoter (<=1kb)
**chr14**	20622759	20623279	521	31	-151.67433815554600	RNASE11	ribonuclease A family member 11 (inactive)	-12875	Distal Intergenic
**chr17**	1977590	1978117	528	37	-149.31855199898300	LOC105371485	uncharacterized LOC105371485	-39599	Intron (ENST00000331238.7/146760, intron 1 of 1)
**chr5**	135028968	135029573	606	47	-148.96881825325800	PITX1	paired like homeodomain 1	2437	Promoter (2-3kb)
**chr19**	56476836	56477397	562	51	-148.59650623155000	ZNF667	zinc finger protein 667	0	Promoter (<=1kb)
**chr5**	135038634	135039049	416	37	-147.50927448354800	EPIST	esophagus epithelial intergenic associated transcript	647	Promoter (<=1kb)
**chr2**	156319556	156320161	606	37	-146.14695619066700	NR4A2	nuclear receptor subfamily 4 group A member 2	8254	Intron (ENST00000634100.1/ENST00000634100.1, intron 1 of 2)
**chr10**	122163185	122163654	470	39	-145.74219295354300	TACC2	transforming acidic coiled-coil containing protein 2	0	Promoter (<=1kb)
**chr22**	46061566	46062090	525	47	145.6786635166690	PRR34-AS1	PRR34 antisense RNA 1	6651	Intron (ENST00000381051.6/124905135, intron 1 of 4)
**chr9**	124450346	124451040	695	42	-145.60654594770000	ADGRD2	adhesion G protein-coupled receptor D2	0	Promoter (<=1kb)
**chr1**	40884325	40884654	330	41	-145.54972242576000	LOC124904157	uncharacterized LOC124904157	20411	Distal Intergenic
**chr12**	130163484	130163863	380	44	-145.38379558049200	FZD10	frizzled class receptor 10	1020	Promoter (1-2kb)
**chr4**	93834341	93835002	662	39	-145.04255219888200	ATOH1	atonal bHLH transcription factor 1	5588	Distal Intergenic
**chr11**	35419764	35420321	558	50	-144.26472441809200	SLC1A2	solute carrier family 1 member 2	0	Promoter (<=1kb)
**chr6**	170287664	170288646	983	40	144.17821700682600	FAM120B	family with sequence similarity 120 member B	-2057	Promoter (2-3kb)
**chr9**	133429619	133430086	468	49	-144.02453370394500	ADAMTS13	ADAM metallopeptidase with thrombospondin type 1 motif 13	7252	3' UTR

**chr4**	41644276	41644655	380	37	-143.91053816201200	LIMCH1	LIM and calponin homology domains 1	30800	Exon (ENST00000513024.5/22998, exon 9 of 26)
**chr10**	27741167	27741805	639	42	-143.64583990653	MKX	mohawk homeobox	1740	Promoter (1-2kb)
**chr10**	102240604	102241291	688	43	-143.56567662581700	PITX3	paired like homeodomain 3	221	Promoter (<=1kb)
**chr12**	114406479	114408138	1660	44	-143.4513245412290	TBX5-AS1	TBX5 antisense RNA 1	0	Promoter (<=1kb)
**chr11**	57482767	57483374	608	36	-143.3703504310990	SLC43A1	solute carrier family 43 member 1	11146	Distal Intergenic
**chr16**	56662786	56663317	532	39	-141.74407500106800	MT1G	metallothionein 1G	4704	Distal Intergenic
**chr1**	110211733	110212171	439	51	-139.52202818657900	KCNC4	potassium voltage-gated channel subfamily C member 4	0	Promoter (<=1kb)
**chr15**	100880461	100880913	453	40	-139.47993154744400	ALDH1A3	aldehyde dehydrogenase 1 family member A3	586	Promoter (<=1kb)
**chr2**	30230560	30231133	574	38	-139.14323992741900	LBH	LBH regulator of WNT signaling pathway	-401	Promoter (<=1kb)
**chr12**	121240583	121240996	414	33	-138.93578894983000	P2RX4	purinergic receptor P2X 4	18251	3' UTR
**chr4**	41878055	41878656	602	35	-138.04116341225100	LINC00682	long intergenic non-protein coding RNA 682	2973	Promoter (2-3kb)
**chr14**	95360397	95360660	264	31	-137.7034270524170	LINC02292	long intergenic non-protein coding RNA 2292	28019	Distal Intergenic
**chr12**	114674486	114674821	336	44	-135.66562709615500	TBX3	T-box transcription factor 3	5112	Exon (ENST00000257566.7/6926, exon 7 of 8)
**chr12**	85278510	85279155	646	38	-134.4346286944150	ALX1	ALX homeobox 1	-1065	Promoter (1-2kb)
**chr3**	61564452	61565013	562	40	-134.10961066906400	PTPRG	protein tyrosine phosphatase receptor type G	2541	Promoter (2-3kb)
**chr4**	153792726	153793262	537	35	-134.00566950124200	SFRP2	secreted frizzled related protein 2	-3643	Distal Intergenic
**chr8**	64587043	64587607	565	36	-132.89472012603800	BHLHE22-AS1	BHLHE22 antisense RNA 1	-5122	Distal Intergenic
**chr16**	54282631	54283236	606	34	-131.5457686881550	IRX3	iroquois homeobox 3	2619	Promoter (2-3kb)
**chr1**	209675533	209676158	626	33	-131.4103524763350	G0S2	G0/G1 switch 2	121	Promoter (<=1kb)
**chr12**	124388969	124389565	597	39	-131.28780792706400	NCOR2	nuclear receptor corepressor 2	-145	Promoter (<=1kb)

**chr17**	35345459	35346066	608	36	-130.96880904223500	SLFN11	schlafen family member 11	16926	Distal Intergenic
**chr5**	93579046	93579586	541	35	-130.34295856282700	NR2F1-AS1	NR2F1 antisense RNA 1	1193	Promoter (1-2kb)
**chr19**	3687983	3688495	513	37	-130.21436134562100	PIP5K1C	phosphatidylinositol-4-phosphate 5-kinase type 1 gamma	11895	Intron (ENST00000679885.1/23396, intron 1 of 18)
**chr17**	61395582	61395988	407	38	-130.1358253176380	TBX2-AS1	TBX2 antisense RNA 1	-135	Promoter (<=1kb)
**chr1**	157194719	157195034	316	34	-130.00502743214200	LOC107985211	uncharacterized LOC107985211	11859	Distal Intergenic
**chr8**	86069324	86069873	550	45	-128.97799174391000	PSKH2	protein serine kinase H2	0	Promoter (<=1kb)
**chr15**	100973701	100974117	417	49	-128.8027687883400	LRRK1	leucine rich repeat kinase 1	-47592	Exon (ENST00000527698.1/79705, exon 3 of 3)
**chr12**	114669395	114670060	666	30	-128.25210269547400	TBX3	T-box transcription factor 3	9873	Distal Intergenic
**chr4**	3746459	3746853	395	34	127.71956514363200	ADRA2C	adrenoceptor alpha 2C	-19532	Distal Intergenic
**chr22**	42283438	42283880	443	33	-127.34272493507800	TCF20	transcription factor 20	117	Promoter (<=1kb)
**chr6**	37649045	37649370	326	43	-127.15760788934500	MDGA1	MAM domain containing glycosylphosphatidylinositol anchor 1	-415	Promoter (<=1kb)
**chr10**	35639589	35639985	397	37	-126.76496639188000	LOC105376494	uncharacterized LOC105376494	-1112	Promoter (1-2kb)
**chr16**	31214782	31215167	386	46	-126.41635101824400	TRIM72	tripartite motif containing 72	663	Promoter (<=1kb)
**chr2**	120741751	120742134	384	42	-126.25737631838100	GLI2	GLI family zinc finger 2	5883	Intron (ENST00000482119.6/2736, intron 1 of 3)
**chr8**	25001357	25001894	538	33	-125.99132797282300	LOC105379330	uncharacterized LOC105379330	9355	Exon (ENST00000670816.1/ENST00000670816.1, exon 2 of 2)
**chr16**	10180229	10180662	434	33	-125.85653515405900	GRIN2A	glutamate ionotropic receptor NMDA type subunit 2A	122	Promoter (<=1kb)
**chr2**	96839483	96839862	380	36	-125.31149508297500	ANKRD23	ankyrin repeat domain 23	1380	Promoter (1-2kb)

**chr15**	45187264	45187722	459	37	-124.37850769459500	SHF	Src homology 2 domain containing F	0	Promoter (<=1kb)
**chr11**	46278120	46278664	545	38	-123.74623663461600	CREB3L1	cAMP responsive element binding protein 3 like 1	458	Promoter (<=1kb)
**chr8**	143770536	143770991	456	36	-123.34947658105400	LOC105375800	SREBF pathway regulator in golgi 1 pseudogene	879	Promoter (<=1kb)
**chr22**	21736212	21736515	304	37	-123.22783076258500	YPEL1	yippee like 1	-418	Promoter (<=1kb)
**chr9**	36739538	36739871	334	33	-122.72855713487200	LOC105376030	uncharacterized LOC105376030	-39604	Distal Intergenic
**chr11**	73309254	73309841	588	31	-122.19223455621200	ARHGEF17-AS1	ARHGEF17 antisense RNA 1	0	Promoter (<=1kb)
**chr3**	194398021	194398459	439	34	-121.83432205711200	GP5	glycoprotein V platelet	807	Promoter (<=1kb)
**chr20**	40690491	40690900	410	42	-121.63932939802300	MAFB	MAF bZIP transcription factor B	-1255	Promoter (1-2kb)
**chr11**	15941267	15941632	366	31	-121.39051339683600	MIR6073	microRNA 6073	27989	Distal Intergenic
**chr15**	100973050	100973395	346	36	-120.63839042075500	LRRK1	leucine rich repeat kinase 1	-48314	Intron (ENST00000527698.1/79705, intron 2 of 2)
**chr11**	31824631	31825302	672	37	-120.17320552205100	PAUPAR	PAX6 upstream antisense RNA	-1045	Promoter (1-2kb)
**chr5**	77628297	77628619	323	37	-119.28871517192800	WDR41	WD repeat domain 41	-7686	Distal Intergenic
**chr7**	128351413	128351725	313	36	-118.9686764925120	RBM28	RNA binding motif protein 28	-7505	Exon (ENST00000489835.6/401399, exon 6 of 6)
**chr11**	69157223	69157723	501	40	-118.70411681475800	SMIM38	small integral membrane protein 38	1288	Promoter (1-2kb)
**chr1**	110067590	110067993	404	30	-118.24728852934700	ALX3	ALX homeobox 3	-2666	Promoter (2-3kb)
**chr10**	75043860	75044098	239	29	-118.00176647574000	KAT6B	lysine acetyltransferase 6B	24222	Exon (ENST00000338487.6/338599, exon 3 of 4)
**chr1**	225677123	225677701	579	32	-117.7796652972060	LOC102723834	uncharacterized LOC102723834	-22563	Distal Intergenic
**chr4**	139735601	139735980	380	38	-117.68103133279600	LOC124900781	uncharacterized LOC124900781	-27073	Intron (ENST00000616265.4/4258, intron 5 of 5)

**chr6**	31730225	31730584	360	31	-117.59867030932400	DDAH2	DDAH family member 2, ADMA-independent	0	Promoter (<=1kb)
**chr2**	232634464	232634908	445	33	-117.3601517742210	EFHD1	EF-hand domain family member D1	748	Promoter (<=1kb)
**chr1**	90726902	90727386	485	33	-117.25336609573500	BARHL2	BarH like homeobox 2	-9600	Distal Intergenic
**chr11**	16610683	16611151	469	31	-115.08776457008200	LOC105376571	uncharacterized LOC105376571	-1981	Promoter (1-2kb)
**chr10**	22252891	22253165	275	33	-114.9189034049330	LOC100130992	uncharacterized LOC100130992	819	Promoter (<=1kb)
**chr15**	66254140	66254861	722	33	-114.34869217066200	MEGF11	multiple EGF like domains 11	-384	Promoter (<=1kb)
**chr1**	161525122	161525478	357	31	-113.98368656892200	HSPA6	heat shock protein family A (Hsp70) member 6	582	Promoter (<=1kb)
**chr17**	6713630	6714015	386	32	-113.74621953873900	SLC13A5	solute carrier family 13 member 5	-253	Promoter (<=1kb)
**chr16**	56675766	56676363	598	35	-112.66455826584800	MT1IP	metallothionein 1I, pseudogene	0	Promoter (<=1kb)
**chr2**	118853091	118853980	890	38	-111.82308666256100	EN1	engrailed homeobox 1	-5443	Distal Intergenic
**chr5**	73435755	73436396	642	32	-110.8149235564430	FOXD1	forkhead box D1	12381	Distal Intergenic
**chr5**	93602307	93602984	678	34	-109.43889103851500	NR2F1	nuclear receptor subfamily 2 group F member 1	10334	Exon (ENST00000623223.1/ENST00000623223.1, exon 1 of 1)
**chr3**	9136212	9136447	236	33	-109.29925852378600	SRGAP3	SLIT-ROBO Rho GTPase activating protein 3	-54925	Intron (ENST00000383836.8/9901, intron 1 of 21)
**chr19**	16325942	16326293	352	28	-109.29677528812900	KLF2	KLF transcription factor 2	1102	Promoter (1-2kb)
**chr6**	37705545	37706087	543	34	-108.8862555356200	MDGA1	MAM domain containing glycosylphosphatidylinositol anchor 1	-6239	Distal Intergenic
**chr11**	61828933	61829340	408	31	-108.5863677596660	FADS1	fatty acid desaturase 1	0	Promoter (<=1kb)
**chr2**	118850060	118850527	468	30	-107.94590670366000	EN1	engrailed homeobox 1	-2412	Promoter (2-3kb)
**chr10**	120949995	120950427	433	25	-107.53505327604900	LOC105378519	uncharacterized LOC105378519	30273	Intron (ENST00000414774.1/105378519, intron 1 of 1)

**chr8**	142873635	142875991	2357	31	107.30394216207700	CYP11B1	cytochrome P450 family 11 subfamily B member 1	0	Promoter (<=1kb)
**chr2**	235671178	235671629	452	35	-107.01892290376400	AGAP1	ArfGAP with GTPase domain, ankyrin repeat and PH domain 1	1574	Promoter (1-2kb)
**chr7**	128201332	128201812	481	36	-106.67565201299300	MIR129-1	microRNA 129-1	-6060	Intron (ENST00000710955.1/124901744, intron 1 of 3)
**chr11**	72241125	72241646	522	33	-106.23019856455200	PHOX2A	paired like homeobox 2A	2530	Promoter (2-3kb)
**chr17**	61456553	61456770	218	35	-106.0799030640230	TBX4	T-box transcription factor 4	89	Promoter (<=1kb)
**chr10**	71396215	71396620	406	34	-105.63417161525300	CDH23	cadherin related 23	-300	Promoter (<=1kb)
**chr12**	49335946	49336272	327	35	-104.53903384186600	C1QL4	complement C1q like 4	916	Promoter (<=1kb)
**chr15**	84079727	84080146	420	32	-104.50191591984600	EFL1P1	elongation factor like GTPase 1 pseudogene 1	-22	Promoter (<=1kb)
**chr22**	42432077	42432646	570	25	-103.15391240967400	NFAM1	NFAT activating protein with ITAM motif 1	0	Promoter (<=1kb)
**chr12**	54417971	54418824	854	26	-103.05776420276100	ITGA5	integrin subunit alpha 5	303	Promoter (<=1kb)
**chr14**	60643566	60644421	856	25	-102.982447695358	SIX1	SIX homeobox 1	5056	3' UTR
**chr17**	29572795	29573130	336	41	-102.62798889085600	GIT1	GIT ArfGAP 1	3001	3' UTR
**chr18**	12307324	12307738	415	26	-102.5767416804970	TUBB6	tubulin beta 6 class V	0	Promoter (<=1kb)
**chr1**	1034010	1034313	304	42	-102.40841239732400	AGRN	agrin	0	Promoter (<=1kb)
**chr8**	59117338	59117846	509	24	-102.30241275163800	TOX-DT	TOX divergent transcript	-1194	Promoter (1-2kb)
**chr4**	1802514	1802998	485	31	101.97383644332300	FGFR3	fibroblast growth factor receptor 3	602	Promoter (<=1kb)
**chr13**	99657881	99658260	380	29	-101.89883138522700	MIR4306	microRNA 4306	14822	Intron (ENST00000376355.7/171425, intron 1 of 7)
**chr8**	143953563	143953922	360	28	-101.53526822397600	PLEC	plectin	0	Promoter (<=1kb)

**chr6**	36285008	36285602	595	28	-101.18820602170700	PNPLA1	patatin like phospholipase domain containing 1	14548	Intron (ENST00000312917.9/285848, intron 1 of 7)
**chr5**	179530334	179530734	401	30	-100.98638028501200	LOC100289470	chromosome 5 open reading frame 60-like	7877	Exon (ENST00000702627.1/ENST00000702627.1, exon 1 of 1)
**chr12**	57631704	57632087	384	29	-100.6720687852190	B4GALNT1	beta-1,4-N-acetyl-galactosaminyltransferase 1	560	Promoter (<=1kb)
**chr1**	39409447	39409900	454	27	-100.51133449221100	MACF1	microtubule actin crosslinking factor 1	0	Promoter (<=1kb)
**chr2**	217756505	217756857	353	28	-100.11644183830600	DIRC3	disrupted in renal carcinoma 3	0	Promoter (<=1kb)
**chr5**	31639687	31640146	460	28	-100.06715129927200	PDZD2	PDZ domain containing 2	556	Promoter (<=1kb)
**chr7**	143885125	143885537	413	27	-99.9421298338588	TCAF1	TRPM8 channel associated factor 1	0	Promoter (<=1kb)
**chr3**	32392372	32392858	487	30	-99.8730390888966	CMTM7	CKLF like MARVEL transmembrane domain containing 7	333	Promoter (<=1kb)
**chr6**	35511573	35511871	299	30	-99.82800824874870	TULP1	TUB like protein 1	96	Promoter (<=1kb)
**chr8**	41567445	41567872	428	29	-99.57746394419450	LOC124901939	uncharacterized LOC124901939	587	Promoter (<=1kb)
**chr7**	155459960	155460322	363	24	-98.9907231485833	EN2	engrailed homeobox 2	1831	Promoter (1-2kb)
**chr9**	127926950	127927351	402	31	-98.66204897635130	PIP5KL1	phosphatidylinositol-4-phosphate 5-kinase like 1	0	Promoter (<=1kb)
**chr2**	237691057	237691440	384	35	-98.60569308308860	LRRFIP1	LRR binding FLII interacting protein 1	-705	Promoter (<=1kb)
**chr2**	237486290	237486699	410	33	-98.5756299983827	MLPH	melanophilin	0	Promoter (<=1kb)
**chr9**	92715062	92715425	364	27	98.23951733379900	IPPK	inositol-pentakisphosphate 2-kinase	-44931	3' UTR
**chr15**	31393003	31393494	492	31	-97.79440977593960	KLF13	KLF transcription factor 13	-81	Promoter (<=1kb)
**chr19**	13938205	13939010	806	24	-97.46566497495550	PODNL1	podocan like 1	0	Promoter (<=1kb)
**chr3**	137032600	137032967	368	22	-97.26726564262930	IL20RB-AS1	IL20RB antisense RNA 1	-50404	Distal Intergenic
**chr2**	176175110	176175710	601	30	-97.0326469037982	HAGLR	HOXD antisense growth-associated long non-coding RNA	1858	Promoter (1-2kb)

**chr6**	108170399	108171233	835	28	-96.82910110104940	NR2E1	nuclear receptor subfamily 2 group E member 1	1636	Promoter (1-2kb)
**chr7**	20776330	20776942	613	29	-96.52208919515340	SP8	Sp8 transcription factor	9944	3' UTR
**chr6**	168442248	168442599	352	25	-95.85837384568910	SMOC2	SPARC related modular calcium binding 2	1064	Promoter (1-2kb)
**chr4**	139279368	139279842	475	25	-95.63713312861020	MGARP	mitochondria localized glutamic acid rich protein	383	Promoter (<=1kb)
**chr3**	138942916	138943592	677	25	-95.27477655892560	LINC01391	long intergenic non-protein coding RNA 1391	-338	Promoter (<=1kb)
**chr1**	165117497	165117850	354	33	-94.54842467815310	LMX1A-AS2	LMX1A antisense RNA 2	-92777	Distal Intergenic
**chr1**	180913260	180913594	335	30	-94.44482916670630	KIAA1614	KIAA1614	106	Promoter (<=1kb)
**chr6**	41371607	41371864	258	28	-94.36895923050910	NCR2	natural cytotoxicity triggering receptor 2	35756	Distal Intergenic
**chr6**	44219428	44219662	235	25	-93.95528571059000	SLC29A1	solute carrier family 29 member 1 (Augustine blood group)	0	Promoter (<=1kb)
**chr7**	102045336	102045596	261	20	-93.39456809041940	CUX1	cut like homeobox 1	-51882	Intron (ENST00000437600.9/1523, intron 3 of 22)
**chr10**	96719774	96720303	530	27	-92.92447126026480	PIK3AP1	phosphoinositide-3-kinase adaptor protein 1	211	Promoter (<=1kb)
**chr10**	16519657	16520019	363	26	-92.6640158463917	C1QL3	complement C1q like 3	1860	Promoter (1-2kb)
**chr1**	229407082	229407517	436	30	-92.40440450973000	LOC124904541	uncharacterized LOC124904541	0	Promoter (<=1kb)
**chr20**	21102002	21102366	365	25	-92.20955509327960	LINC00237	long intergenic non-protein coding RNA 237	17	Promoter (<=1kb)
**chr17**	6714049	6714308	260	22	-91.79147458800520	SLC13A5	solute carrier family 13 member 5	-672	Promoter (<=1kb)
**chr3**	125357331	125357869	539	26	-91.77336384176940	ZNF148	zinc finger protein 148	0	Promoter (<=1kb)
**chr14**	104179157	104179621	465	24	91.42865173044340	KIF26A	kinesin family member 26A	40117	Exon (ENST00000697132.1/26153, exon 14 of 15)
**chr6**	29975378	29975900	523	27	-90.40751409160350	HCG9	HLA complex group 9	266	Promoter (<=1kb)
**chr6**	111566863	111567455	593	19	-89.90759839028240	TRAF3IP2	TRAF3 interacting protein 2	0	Promoter (<=1kb)
**chr5**	116576498	116577247	750	31	-89.7273231827311	SEMA6A-AS2	SEMA6A antisense RNA 2	1343	Promoter (1-2kb)
**chr6**	100458507	100459019	513	30	-89.67971646499670	SIM1	SIM bHLH transcription factor 1	4658	Intron (ENST00000369208.8/6492, intron 2 of 11)

**chr11**	134277016	134277490	475	26	-89.42581712035310	GLB1L3	galactosidase beta 1 like 3	637	Promoter (<=1kb)
**chr19**	10286885	10287293	409	31	-88.45189818906710	ICAM4	intercellular adhesion molecule 4 (Landsteiner-Wiener blood group)	0	Promoter (<=1kb)
**chr14**	100307185	100307503	319	26	-88.12352799278900	MIR345	microRNA 345	-356	Promoter (<=1kb)
**chr15**	48646068	48646360	293	31	-88.0454004937391	FBN1-DT	FBN1 divergent transcript	117	Promoter (<=1kb)
**chr13**	99967922	99968285	364	26	-87.78509547246390	ZIC5	Zic family member 5	3482	Intron (ENST00000267294.5/85416, intron 1 of 1)
**chr9**	137434959	137435310	352	26	87.7712540649462	ENTPD8	ectonucleoside triphosphate diphosphohydrolase 8	2936	Promoter (2-3kb)
**chr19**	35704223	35704572	350	24	-87.39482313608160	ZBTB32	zinc finger and BTB domain containing 32	0	Promoter (<=1kb)
**chr2**	172081433	172081963	531	23	-87.10202056349040	DLX1	distal-less homeobox 1	-2777	Promoter (2-3kb)
**chr2**	221570379	221570695	317	19	-87.04054785286020	EPHA4	EPH receptor A4	-898	Promoter (<=1kb)
**chr12**	124457235	124457691	457	21	-85.63099371108370	NCOR2	nuclear receptor corepressor 2	26078	Intron (ENST00000404621.5/9612, intron 6 of 46)
**chr1**	44786356	44786641	286	30	-85.02973647046230	BEST4	bestrophin 4	1529	Promoter (1-2kb)
**chr2**	170717263	170717631	369	26	-84.51511224367100	SP5	Sp5 transcription factor	1221	Promoter (1-2kb)
**chr5**	81233144	81233557	414	27	-84.48845231823630	CKMT2	creatine kinase, mitochondrial 2	0	Promoter (<=1kb)
**chr11**	63769707	63770037	331	23	-84.3521950239669	ZFTA	zinc finger translocation associated	-932	Promoter (<=1kb)
**chr11**	7252767	7253003	237	24	-84.08762681813690	SYT9	synaptotagmin 9	597	Promoter (<=1kb)
**chr5**	7395261	7395641	381	23	-83.98254917106680	ADCY2	adenylate cyclase 2	-497	Promoter (<=1kb)
**chr15**	45704142	45704622	481	31	-83.86983228270550	LOC105370802	uncharacterized LOC105370802	-456	Promoter (<=1kb)
**chr14**	36516149	36516951	803	17	-83.62034832356720	SFTA3	surfactant associated 3	-2320	Promoter (2-3kb)
**chr1**	161524898	161525105	208	21	-83.43238051094750	HSPA6	heat shock protein family A (Hsp70) member 6	358	Promoter (<=1kb)
**chr1**	170666812	170667097	286	21	-83.34985881749350	PRRX1	paired related homeobox 1	2681	Promoter (2-3kb)
**chr19**	48415934	48416154	221	23	-83.32749482245220	GRIN2D	glutamate ionotropic receptor NMDA type subunit 2D	22266	Exon (ENST00000263269.4/2906, exon 8 of 14)
**chr18**	12420824	12421134	311	20	-83.01603417831820	PRELID3A	PRELI domain containing 3A	-152	Promoter (<=1kb)

**chr11**	57349853	57350192	340	21	-82.95897823419250	P2RX3	purinergic receptor P2X 3	2429	Promoter (2-3kb)
**chr19**	35557567	35557833	267	22	-82.84508334763820	ATP4A	ATPase H+/K+ transporting subunit alpha	0	Promoter (<=1kb)
**chr14**	56807567	56807994	428	26	-82.68523267342790	OTX2	orthodenticle homeobox 2	-1900	Promoter (1-2kb)
**chr9**	99825425	99825816	392	23	-82.63166844785330	NR4A3	nuclear receptor subfamily 4 group A member 3	-911	Promoter (<=1kb)
**chr14**	74923038	74923284	247	29	-82.52627325879110	RPS6KL1	ribosomal protein S6 kinase like 1	0	Promoter (<=1kb)
**chr5**	128536888	128537158	271	27	-82.35397884163670	FBN2	fibrillin 2	-443	Promoter (<=1kb)
**chr7**	152924832	152925285	454	26	-82.2661632087486	ACTR3B	actin related protein 3B	114090	Exon (ENST00000658865.1/ENST00000658865.1, exon 1 of 4)
**chr20**	50172361	50172639	279	22	-81.78413405609930	LINC01273	long intergenic non-protein coding RNA 1273	0	Promoter (<=1kb)
**chr16**	68448771	68449118	348	22	-81.70566103426750	LOC124903705	uncharacterized LOC124903705	0	Promoter (<=1kb)
**chr2**	72925442	72925874	433	26	-81.61033036618680	EMX1	empty spiracles homeobox 1	1454	Promoter (1-2kb)
**chr2**	74555236	74555534	299	28	-81.43686619180980	DOK1	docking protein 1	30	Promoter (<=1kb)
**chr16**	56638366	56638648	283	29	-81.42373902473700	MT1A	metallothionein 1A	-18	Promoter (<=1kb)
**chr12**	15221053	15221308	256	20	-81.07146137430900	RERG	RAS like estrogen regulated growth inhibitor	0	Promoter (<=1kb)
**chr4**	1309215	1309869	655	24	-81.04230152549020	MAEA	macrophage erythroblast attacher, E3 ubiquitin ligase	0	Promoter (<=1kb)
**chr3**	139538768	139539167	400	23	-81.0297550536133	RBP1	retinol binding protein 1	469	Promoter (<=1kb)
**chr1**	51729875	51730148	274	23	-81.00852040352700	OSBPL9	oxysterol binding protein like 9	0	Promoter (<=1kb)
**chr4**	152045706	152046144	439	23	-80.87735879957470	LINC02273	long intergenic non-protein coding RNA 2273	-44315	Distal Intergenic
**chr6**	157236675	157236962	288	34	-80.21076576618540	ARID1B	AT-rich interaction domain 1B	38166	Distal Intergenic
**chr9**	14345873	14346148	276	24	-80.09402832196180	NFIB-AS1	NFIB antisense RNA 1	-1172	Promoter (1-2kb)
**chr3**	71305307	71306152	846	21	-79.80395231537600	FOXP1	forkhead box P1	0	Promoter (<=1kb)
**chr8**	41310141	41310817	677	21	-79.55144909507400	SFRP1	secreted frizzled related protein 1	-668	Promoter (<=1kb)
**chr22**	23838387	23838604	218	21	-79.44918626694890	DERL3	derlin 3	-184	Promoter (<=1kb)

**chr2**	158967751	158968191	441	20	-79.22557682086650	TANC1	tetratricopeptide repeat, ankyrin repeat and coiled-coil containing 1	-449	Promoter (<=1kb)
**chr9**	136404751	136405337	587	23	79.21426005757520	ENTR1	endosome associated trafficking regulator 1	0	Promoter (<=1kb)
**chr10**	101132634	101133085	452	23	-79.14195029118750	TLX1	T cell leukemia homeobox 1	0	Promoter (<=1kb)
**chr5**	93571238	93571580	343	19	-78.83990239015360	NR2F1-AS1	NR2F1 antisense RNA 1	0	Promoter (<=1kb)
**chr19**	40365711	40366115	405	21	-78.83731945860490	PLD3	phospholipase D family member 3	0	Promoter (<=1kb)
**chr14**	53952754	53953148	395	24	-78.08065832925390	BMP4	bone morphogenetic protein 4	281	Promoter (<=1kb)
**chr14**	94771645	94771897	253	18	-77.59993927087050	GSC	goosecoid homeobox	-1532	Promoter (1-2kb)
**chr15**	78619905	78620177	273	22	-77.39763464550460	CHRNA3	cholinergic receptor nicotinic alpha 3 subunit	84	Promoter (<=1kb)
**chr6**	5999941	6000417	477	22	-77.25098006854760	NRN1	neuritin 1	2546	Promoter (2-3kb)
**chr16**	56632381	56632641	261	28	-77.21532001753760	MT1M	metallothionein 1M	-18	Promoter (<=1kb)
**chr10**	99537682	99537963	282	22	-77.08528128601630	NKX2-3	NK2 homeobox 3	4740	Distal Intergenic
**chr2**	70906855	70907394	540	27	-76.58582684709180	ATP6V1B1	ATPase H+ transporting V1 subunit B1	6057	Intron (ENST00000234392.3/25806, intron 1 of 2)
**chr12**	53949722	53950063	342	25	-76.36144377911760	HOXC12	homeobox C12	-4840	Distal Intergenic
**chr7**	44324095	44324652	558	20	-76.24451441133550	CAMK2B	calcium/calmodulin dependent protein kinase II beta	-133	Promoter (<=1kb)
**chr20**	63401870	63402287	418	23	75.8353470576256	KCNQ2	potassium voltage-gated channel subfamily Q member 2	5725	3' UTR
**chr19**	35756709	35756948	240	21	-75.59031939824130	HSPB6	heat shock protein family B (small) member 6	61	Promoter (<=1kb)
**chr11**	61534632	61535024	393	20	75.53036275883320	SYT7	synaptotagmin 7	-1529	Promoter (1-2kb)
**chr12**	34338072	34338335	264	22	75.42041905151850	NA	NA	-118826	Distal Intergenic
**chr5**	2007497	2007801	305	22	75.3683540331537	LOC124901165	keratin-associated protein 5-5-like	58550	Intron (ENST00000653000.1/105374618, intron 2 of 8)
**chr8**	80565910	80566111	202	20	-75.11519574383850	ZNF704	zinc finger protein 704	74584	Distal Intergenic
**chr3**	65597536	65598069	534	27	-74.99043297589810	MAGI1	membrane associated guanylate kinase, WW and PDZ domain containing 1	0	Promoter (<=1kb)

**chr16**	56193644	56194087	444	22	-74.74938968208580	GNAO1	G protein subunit alpha o1	0	Promoter (<=1kb)
**chr17**	4641737	4642202	466	24	-74.73312453860250	ALOX15	arachidonate 15-lipoxygenase	0	Promoter (<=1kb)
**chr9**	121698493	121698805	313	21	-74.21803899156400	DAB2IP	DAB2 interacting protein	-1754	Promoter (1-2kb)
**chr19**	49130417	49130688	272	22	74.21640634399480	PPFIA3	PTPRF interacting protein alpha 3	1837	Promoter (1-2kb)
**chr20**	63641331	63641637	307	27	74.178961793534	STMN3	stathmin 3	10757	3' UTR
**chr12**	106139836	106140137	302	20	-73.97253571525250	NUAK1	NUAK family kinase 1	-882	Promoter (<=1kb)
**chr16**	85171982	85172549	568	21	-73.58711358281200	GSE1	Gse1 coiled-coil protein	706	Promoter (<=1kb)
**chr9**	130666762	130666971	210	24	-73.39367992705290	PRDM12	PR/SET domain 12	2168	Promoter (2-3kb)
**chr6**	37536458	37536792	335	18	-73.33668679639940	MIR4462	microRNA 4462	18630	Downstream (<=300bp)
**chr6**	21597730	21598912	1183	18	-73.12338057080740	LOC107986579	uncharacterized LOC107986579	-2137	Promoter (2-3kb)
**chr18**	12287250	12287520	271	25	-72.82282350705240	TUBB6	tubulin beta 6 class V	-20149	Distal Intergenic
**chr3**	69385691	69386021	331	25	-72.71299059761750	FRMD4B	FERM domain containing 4B	0	Promoter (<=1kb)
**chr9**	126222885	126223240	356	22	-72.67918528497240	LOC101929116	uncharacterized LOC101929116	52678	Distal Intergenic
**chr9**	21989805	21990108	304	22	-72.50708038455180	CDKN2B-AS1	CDKN2B antisense RNA 1	-4031	Exon (ENST00000470819.2/1029, exon 3 of 3)
**chr17**	74359919	74360234	316	21	72.39219853737190	BTBD17	BTB domain containing 17	1634	Promoter (1-2kb)
**chr4**	84483834	84484144	311	21	-72.35793688343930	NKX6-1	NK6 homeobox 1	12806	Distal Intergenic
**chr11**	68128535	68128838	304	22	-72.32680827380250	CHKA-DT	CHKA divergent transcript	6482	Intron (ENST00000530842.2/105369362, intron 1 of 2)
**chr3**	136818718	136818956	239	17	-72.31931915435740	SLC35G2	solute carrier family 35 member G2	71	Promoter (<=1kb)
**chr5**	36692925	36693242	318	23	-72.31000892201610	SLC1A3	solute carrier family 1 member 3	9578	Intron (ENST00000510740.1/107986412, intron 1 of 2)
**chr15**	40405879	40406227	349	26	-72.25024151897930	IVD	isovaleryl-CoA dehydrogenase	0	Promoter (<=1kb)
**chr9**	32955715	32955983	269	23	-72.23046742370890	APTX	aprataxin	19918	Intron (ENST00000672846.1/54840, intron 9 of 10)
**chr9**	20619306	20620186	881	18	-72.17754618307100	MLLT3	MLLT3 super elongation complex subunit	1687	Promoter (1-2kb)

**chr1**	197921329	197921614	286	23	-72.1361310870613	LHX9	LIM homeobox 9	3957	Exon (ENST00000367391.5/56956, exon 4 of 6)
**chr17**	72115812	72116068	257	20	-71.50855952485360	SOX9-AS1	SOX9 antisense RNA 1	0	Promoter (<=1kb)
**chr2**	200586436	200586819	384	20	-71.46975025426540	AOX1	aldehyde oxidase 1	422	Promoter (<=1kb)
**chr13**	74132786	74133263	478	21	-71.3513422629699	KLF12	KLF transcription factor 12	666	Promoter (<=1kb)
**chr9**	128703167	128703478	312	25	-71.21914236019450	PKN3	protein kinase N3	664	Promoter (<=1kb)
**chr4**	54656941	54657407	467	22	-71.1277293960282	KIT	KIT proto-oncogene, receptor tyrosine kinase	0	Promoter (<=1kb)
**chr2**	172674737	172675076	340	19	-71.06582939310480	LOC124906146	small nucleolar RNA U13	-16223	Exon (ENST00000455767.1/ENST00000455767.1, exon 1 of 1)
**chr19**	58347415	58347616	202	29	-71.0547625387892	A1BG	alpha-1-B glycoprotein	18	Promoter (<=1kb)
**chr7**	129824745	129825118	374	18	-70.92906669917590	UBE2H	ubiquitin conjugating enzyme E2 H	14623	Distal Intergenic
**chr11**	59555750	59556036	287	17	-70.17136611516900	LOC101927226	uncharacterized LOC101927226	-9887	Intron (ENST00000659845.1/102723575, intron 1 of 1)
**chr19**	1775188	1775403	216	21	-70.13076591931910	ATP8B3	ATPase phospholipid transporting 8B3	15414	Exon (ENST00000382349.5/390874, exon 2 of 2)
**chr15**	96353746	96354071	326	23	-70.12201700475930	LOC101927263	uncharacterized LOC101927263	-166	Promoter (<=1kb)
**chr19**	49142836	49143050	215	19	-69.96188911852990	PPFIA3	PTPRF interacting protein alpha 3	0	Promoter (<=1kb)
**chr12**	53961489	53961797	309	25	-69.83847976578330	HOTAIR	HOX transcript antisense RNA	4610	Distal Intergenic
**chr4**	2416584	2416857	274	20	-69.78474064652270	ZFYVE28	zinc finger FYVE-type containing 28	1752	Promoter (1-2kb)
**chr5**	2754398	2754829	432	22	-69.74911705290290	IRX2-DT	IRX2 divergent transcript	2133	Promoter (2-3kb)
**chr1**	156245611	156245812	202	23	-69.68014759331020	PAQR6	progestin and adipoQ receptor family member 6	2202	Promoter (2-3kb)
**chr2**	241200224	241200561	338	23	-69.55593001876830	ANO7	anoctamin 7	1127	Promoter (1-2kb)
**chr17**	48587000	48590117	3118	21	-69.55320396857450	HOXB3	homeobox B3	118	Promoter (<=1kb)
**chr19**	46413546	46413925	380	23	-68.63141759822170	CCDC8	coiled-coil domain containing 8	0	Promoter (<=1kb)
**chr8**	85438049	85438478	430	20	-68.0859843671555	CA3	carbonic anhydrase 3	-380	Promoter (<=1kb)
**chr2**	74499701	74499910	210	21	-67.59469954951080	LBX2	ladybird homeobox 2	-98	Promoter (<=1kb)

**chr20**	21702184	21702594	411	20	-67.54283892221180	LINC01726	long intergenic non-protein coding RNA 1726	991	Promoter (<=1kb)
**chr2**	19358818	19359128	311	22	-67.26887274485940	OSR1	odd-skipped related transcription factor 1	-195	Promoter (<=1kb)
**chr2**	63059331	63059586	256	20	-66.99070384828000	OTX1	orthodenticle homeobox 1	8480	Distal Intergenic
**chr10**	19488788	19489132	345	18	-66.06513658403290	MALRD1	MAM and LDL receptor class A domain containing 1	0	Promoter (<=1kb)
**chr4**	112511295	112511545	251	20	-65.9427503432025	LOC124900760	uncharacterized LOC124900760	-3840	Distal Intergenic
**chr11**	65833108	65833440	333	22	-65.46041499886980	SNX32	sorting nexin 32	-394	Promoter (<=1kb)
**chr6**	39228295	39228633	339	21	65.3057997425362	KCNK5	potassium two pore domain channel subfamily K member 5	842	Promoter (<=1kb)
**chr10**	101237873	101238386	514	19	-65.23924969825530	LBX1-AS1	LBX1 antisense RNA 1	1805	Promoter (1-2kb)
**chr2**	37672686	37673158	473	18	-65.07554799697670	CDC42EP3	CDC42 effector protein 3	-151	Promoter (<=1kb)
**chr22**	20438195	20438533	339	22	-64.54975230532920	SCARF2	scavenger receptor class F member 2	-369	Promoter (<=1kb)
**chr1**	9183119	9183350	232	24	-64.33281182653000	MIR34AHG	MIR34A host gene	-727	Promoter (<=1kb)
**chr15**	89377521	89377749	229	19	-64.06730675195270	MIR9-3HG	MIR9-3 host gene	0	Promoter (<=1kb)
**chr9**	87498998	87499299	302	17	-63.73181625351090	DAPK1	death associated protein kinase 1	0	Promoter (<=1kb)
**chr6**	168384490	168384952	463	20	-63.646361343604800	SMOC2	SPARC related modular calcium binding 2	-56199	Distal Intergenic
**chr7**	157675390	157675791	402	20	63.62716163214070	PTPRN2	protein tyrosine phosphatase receptor type N2	-53881	Intron (ENST00000389413.7/5799, intron 12 of 21)
**chr8**	144532859	144533344	486	22	63.53098485619320	C8orf82	chromosome 8 open reading frame 82	-3727	Exon (ENST00000276826.5/80728, exon 7 of 10)
**chr9**	107491257	107491560	304	21	-63.44714423786750	KLF4	KLF transcription factor 4	-775	Promoter (<=1kb)
**chr12**	114691437	114692152	716	19	-63.20089606910060	TBX3	T-box transcription factor 3	-7262	Intron (ENST00000660721.1/ENST00000660721.1, intron 1 of 1)
**chr8**	10731428	10731642	215	18	-63.15646885262610	SOX7	SRY-box transcription factor 7	-917	Promoter (<=1kb)
**chr1**	1430627	1430933	307	17	-63.06766483951400	LINC01770	long intergenic non-protein coding RNA 1770	910	Promoter (<=1kb)
**chr17**	63700937	63701190	254	22	-62.90706888544790	LIMD2	LIM domain containing 2	0	Promoter (<=1kb)

**chr14**	70186817	70187202	386	18	-62.83462489610100	LOC646548	ADAM metallopeptidase domain 20 pseudogene	0	Promoter (<=1kb)
**chr10**	100347827	100348175	349	21	-62.766426354034200	SCD	stearoyl-CoA desaturase	594	Promoter (<=1kb)
**chr12**	114694937	114695858	922	12	-62.75235181803160	TBX3	T-box transcription factor 3	-10762	Intron (ENST00000660721.1/ENST00000660721.1, intron 1 of 1)
**chr7**	2477616	2477870	255	16	-62.58199429897400	GRIFIN	galectin-related inter-fiber protein	-722	Promoter (<=1kb)
**chr19**	18304836	18305322	487	17	-62.35293816232090	LSM 4.00	LSM4 homolog, U6 small nuclear RNA and mRNA degradation associated	10937	Distal Intergenic
**chr6**	108167547	108167860	314	20	-61.78009289559080	NR2E1	nuclear receptor subfamily 2 group E member 1	-163	Promoter (<=1kb)
**chr2**	176171527	176171760	234	20	-61.72218258009970	HAGLR	HOXD antisense growth-associated long non-coding RNA	5808	Exon (ENST00000683222.1/3232, exon 4 of 4)
**chr13**	113795066	113795410	345	19	61.546696136902100	TMEM255B	transmembrane protein 255B	-4478	Exon (ENST00000375353.5/348013, exon 4 of 9)
**chr10**	100737253	100737615	363	19	-61.18953158158110	PAX2	paired box 2	1650	Promoter (1-2kb)
**chr7**	128853908	128854204	297	21	60.68068345853020	FLNC-AS1	FLNC antisense RNA 1	8422	Exon (ENST00000325888.13/2318, exon 40 of 48)
**chr15**	64776448	64776713	266	19	-60.42576879482790	RBPMS2	RNA binding protein, mRNA processing factor 2	-859	Promoter (<=1kb)
**chr10**	117136528	117136852	325	20	-60.35376904760560	VAX1	ventral anterior homeobox 1	1418	Promoter (1-2kb)
**chr1**	26360587	26360789	203	18	-60.29280024523130	CRYBG2	crystallin beta-gamma domain containing 2	-507	Promoter (<=1kb)
**chr8**	38770371	38770587	217	16	-60.20807173057620	TACC1	transforming acidic coiled-coil containing protein 1	13072	Intron (ENST00000519416.5/6867, intron 1 of 12)
**chr9**	79570158	79570461	304	16	-60.10169043506180	TLE4	TLE family member 4, transcriptional corepressor	-1312	Promoter (1-2kb)
**chr12**	132407059	132407404	346	18	60.03640314117380	LOC101928416	uncharacterized LOC101928416	77209	Distal Intergenic
**chr6**	6004664	6004864	201	20	-59.68222830736420	NRN1	neuritin 1	-620	Promoter (<=1kb)
**chr17**	8002522	8002780	259	15	-59.28598450433880	GUCY2D	guanylate cyclase 2D, retinal	0	Promoter (<=1kb)

**chr2**	238231241	238231466	226	19	-58.923345419735300	LINC02610	long intergenic non-protein coding RNA 2610	0	Promoter (<=1kb)
**chr10**	99533769	99534135	367	17	-58.88082530155900	NKX2-3	NK2 homeobox 3	827	Promoter (<=1kb)
**chr20**	45100994	45101228	235	22	-58.796170081645000	KCNS1	potassium voltage-gated channel modifier subfamily S member 1	0	Promoter (<=1kb)
**chr5**	138139149	138139488	340	17	-58.77812467012040	NME5	NME/NM23 family member 5	0	Promoter (<=1kb)
**chr10**	26931305	26931516	212	14	-58.62709917941930	FAM238C	family with sequence similarity 238 member C	3000	Promoter (2-3kb)
**chr14**	36659891	36660252	362	16	-58.56860656468090	LOC105370455	uncharacterized LOC105370455	-1090	Promoter (1-2kb)
**chr8**	10731131	10731385	255	19	-58.48558955599470	SOX7	SRY-box transcription factor 7	-620	Promoter (<=1kb)
**chr21**	36702178	36702818	641	14	-58.064059061207	SIM2	SIM bHLH transcription factor 2	3044	Intron (ENST00000290399.11/6493, intron 1 of 10)
**chr10**	101587379	101587836	458	15	-57.8818931387599	POLL	DNA polymerase lambda	0	Promoter (<=1kb)
**chr17**	33013592	33013803	212	12	57.85012831679250	SPACA3	sperm acrosome associated 3	20741	3' UTR
**chr3**	138940023	138940264	242	21	-57.81193935698340	LINC01391	long intergenic non-protein coding RNA 1391	2314	Promoter (2-3kb)
**chr17**	82985975	82986358	384	17	-57.750899007933300	B3GNTL1	UDP-GlcNAc:betaGal beta-1,3-N-acetylglucosaminyltransferase like 1	20630	Intron (ENST00000320865.4/146712, intron 6 of 12)
**chr14**	104905192	104905907	716	18	57.52443122566890	CEP170B	centrosomal protein 170B	11258	Exon (ENST00000555713.1/ENST00000555713.1, exon 3 of 3)
**chr7**	1144440	1144709	270	17	57.490038799289700	C7orf50	chromosome 7 open reading frame 50	-6180	Distal Intergenic
**chr2**	74148342	74148563	222	14	-57.31218614262670	BOLA3-DT	BOLA3 divergent transcript	263	Promoter (<=1kb)
**chr2**	15942863	15943196	334	14	-57.046022748738300	MYCN	MYCN proto-oncogene, bHLH transcription factor	-361	Promoter (<=1kb)
**chr2**	74415569	74415869	301	19	-56.884218739159200	C2orf81	chromosome 2 open reading frame 81	1848	Promoter (1-2kb)
**chr17**	43530173	43531114	942	14	-56.7693170109523	ETV4	ETS variant transcription factor 4	0	Promoter (<=1kb)
**chr2**	118856991	118857234	244	18	-56.72701728189640	EN1	engrailed homeobox 1	-9343	Distal Intergenic

**chr19**	7874705	7875019	315	19	-56.62243959334150	PRR36	proline rich 36	-315	Promoter (<=1kb)
**chr11**	62688119	62688377	259	23	-56.499270927960200	LRRN4CL	LRRN4 C-terminal like	1153	Promoter (1-2kb)
**chr12**	114696387	114696789	403	19	-56.42423103152830	TBX3	T-box transcription factor 3	-12212	Intron (ENST00000660721.1/ENST00000660721.1, intron 1 of 1)
**chr14**	36661152	36661397	246	16	-56.29485930240930	PAX9	paired box 9	-463	Promoter (<=1kb)
**chr17**	58487707	58487925	219	15	-55.22952326398910	HSF5	heat shock transcription factor 5	483	Promoter (<=1kb)
**chr2**	133265882	133266288	407	20	-54.810408019645600	NCKAP5-AS2	NCKAP5 antisense RNA 2	0	Promoter (<=1kb)
**chr4**	11427265	11427587	323	18	-54.75621548896590	HS3ST1	heparan sulfate-glucosamine 3-sulfotransferase 1	1010	Promoter (1-2kb)
**chr19**	18595083	18595336	254	19	-54.67344165940330	CRLF1	cytokine receptor like factor 1	-648	Promoter (<=1kb)
**chr2**	19355942	19356185	244	15	-54.585522261410800	OSR1	odd-skipped related transcription factor 1	727	Promoter (<=1kb)
**chr10**	101140156	101140358	203	19	-53.7952999328579	TLX1	T cell leukemia homeobox 1	5842	Distal Intergenic
**chr4**	1798705	1799514	810	20	53.72165946869300	FGFR3	fibroblast growth factor receptor 3	-1948	Promoter (1-2kb)
**chr1**	211829421	211829709	289	16	-53.644823888352500	LPGAT1-AS1	LPGAT1 antisense RNA 1	0	Promoter (<=1kb)
**chr15**	52806209	52806561	353	19	-53.445478949451100	LOC101928499	uncharacterized LOC101928499	-237	Promoter (<=1kb)
**chr5**	125200442	125200894	453	13	-53.342924956057800	LOC101927421	uncharacterized LOC101927421	-124456	Intron (ENST00000647105.1/101927421, intron 2 of 6)
**chr18**	37253374	37253690	317	18	-53.23893913844050	CELF4	CUGBP Elav-like family member 4	291	Promoter (<=1kb)
**chr19**	2281971	2282224	254	16	-53.05637934624140	PEAK3	PEAK family member 3	0	Promoter (<=1kb)
**chr2**	232890961	232891377	417	15	-52.953804125290200	NGEF	neuronal guanine nucleotide exchange factor	0	Promoter (<=1kb)
**chr14**	60637195	60637399	205	13	-52.49430470169220	SIX1	SIX homeobox 1	12078	Distal Intergenic
**chr19**	43580089	43580810	722	15	-52.3926255520341	PINLYP	phospholipase A2 inhibitor and LY6/PLAUR domain containing	0	Promoter (<=1kb)
**chr10**	103660634	103660960	327	16	-52.255935937351900	SH3PXD2A	SH3 and PX domains 2A	744	Promoter (<=1kb)

**chr6**	52307125	52307337	213	18	-52.001409956517000	MCM3	minichromosome maintenance complex component 3	-22244	Distal Intergenic
**chr17**	45222459	45222713	255	14	-51.98959750667450	FMNL1-DT	FMNL1 divergent transcript	-185	Promoter (<=1kb)
**chr16**	55480344	55480597	254	17	-51.901902258380700	MMP2	matrix metallopeptidase 2	0	Promoter (<=1kb)
**chr6**	75202526	75202995	470	17	-51.58848550238870	COL12A1	collagen type XII alpha 1 chain	0	Promoter (<=1kb)
**chr11**	1881699	1882050	352	20	-51.3671177195575	LSP1	lymphocyte specific protein 1	1022	Promoter (1-2kb)
**chr7**	27213690	27213972	283	18	-51.23096800378660	HOTTIP	HOXA distal transcript antisense RNA	11846	Distal Intergenic
**chr16**	29074900	29075149	250	15	-51.226391404683900	RRN3P2	RRN3 pseudogene 2	0	Promoter (<=1kb)
**chr19**	11243220	11243546	327	17	-50.797858943919200	ANGPTL8	angiopoietin like 8	3323	Exon (ENST00000294618.12/57572, exon 12 of 48)
**chr17**	50558785	50559019	235	15	-50.6888860091223	CACNA1G-AS1	CACNA1G antisense RNA 1	391	Promoter (<=1kb)
**chr12**	53751861	53752097	237	15	-50.61964503191160	CISTR	chondrogenesis-associated transcript	0	Promoter (<=1kb)
**chr13**	27971094	27971470	377	17	-50.54407767771200	CDX2	caudal type homeobox 2	-1779	Promoter (1-2kb)
**chr4**	145935593	145935924	332	12	-50.1161458331387	ZNF827	zinc finger protein 827	265	Promoter (<=1kb)
**chr11**	68128171	68128406	236	17	-50.10859447687070	CHKA-DT	CHKA divergent transcript	6118	Intron (ENST00000530842.2/105369362, intron 1 of 2)
**chr2**	199461998	199462204	207	17	-50.08918698789520	SATB2	SATB homeobox 2	474	Promoter (<=1kb)
**chr5**	139481189	139481672	484	15	-49.999517493142900	STING1	stimulator of interferon response cGAMP interactor 1	0	Promoter (<=1kb)
**chr9**	124267254	124267546	293	13	-49.70940888352430	NEK6	NIMA related kinase 6	4378	Intron (ENST00000540326.5/10783, intron 1 of 9)
**chr22**	40679424	40679729	306	17	-49.572887091432900	MCHR1	melanin concentrating hormone receptor 1	0	Promoter (<=1kb)
**chr1**	18630121	18630453	333	18	-49.22539771783150	PAX7	paired box 7	-393	Promoter (<=1kb)
**chr2**	156330090	156330357	268	13	-48.75146810377210	NR4A2	nuclear receptor subfamily 4 group A member 2	0	Promoter (<=1kb)
**chr5**	135489838	135490106	269	13	-48.501493701967100	SLC25A48	solute carrier family 25 member 48	12925	Intron (ENST00000698884.1/153328, intron 3 of 5)
**chr1**	76868815	76869081	267	13	-48.230015698266900	ST6GALNAC5	ST6 N-acetylgalactosaminide alpha-2,6-sialyltransferase 5	1333	Promoter (1-2kb)

**chr6**	137492549	137492997	449	12	-48.17666816195260	OLIG3	oligodendrocyte transcription factor 3	1397	Promoter (1-2kb)
**chr5**	2753603	2753960	358	12	-48.05851327028190	IRX2-DT	IRX2 divergent transcript	1338	Promoter (1-2kb)
**chr2**	96538451	96538667	217	14	-47.95238192141190	ARID5A	AT-rich interaction domain 5A	1106	Promoter (1-2kb)
**chr18**	22167594	22167796	203	15	-47.869394666752000	GATA6-AS1	GATA6 antisense RNA 1 (head to head)	1172	Promoter (1-2kb)
**chr13**	95643597	95643913	317	15	-47.86391539236550	DZIP1	DAZ interacting zinc finger protein 1	-334	Promoter (<=1kb)
**chr6**	28400087	28400324	238	11	-47.845581141275600	ZSCAN12	zinc finger and SCAN domain containing 12	-340	Promoter (<=1kb)
**chr1**	226737145	226737392	248	13	-47.535752292384000	ITPKB	inositol-trisphosphate 3-kinase B	437	Promoter (<=1kb)
**chr5**	173242474	173243062	589	16	-47.30061068595480	NKX2-5	NK2 homeobox 5	-7163	Distal Intergenic
**chr10**	124450633	124451672	1040	14	-47.28971498013150	NKX1-2	NK1 homeobox 2	-598	Promoter (<=1kb)
**chr16**	23694921	23695216	296	13	-47.226650379620600	ERN 2.00	endoplasmic reticulum to nucleus signaling 2	-161	Promoter (<=1kb)
**chr14**	51095897	51096172	276	13	-47.160771005195600	TRIM9	tripartite motif containing 9	0	Promoter (<=1kb)
**chr15**	52529730	52530089	360	13	-47.131562940239700	MYO5A	myosin VA	-598	Promoter (<=1kb)
**chr11**	66048848	66049097	250	17	-47.11934908837550	GAL3ST3	galactose-3-O-sulfotransferase 3	54	Promoter (<=1kb)
**chr14**	21069139	21069417	279	14	-47.04236117664840	ARHGEF40	Rho guanine nucleotide exchange factor 40	-856	Promoter (<=1kb)
**chr12**	54216086	54216390	305	14	-46.97231796838790	MIR3198-2	microRNA 3198-2	15086	Distal Intergenic
**chr16**	87692287	87692959	673	14	46.913762558121400	LOC100129215	uncharacterized LOC100129215	3188	Intron (ENST00000537256.5/57338, intron 4 of 5)
**chr13**	114005019	114005234	216	13	46.844598871773500	C13orf46	chromosome 13 open reading frame 46	-30943	Intron (ENST00000334062.8/22821, intron 18 of 23)
**chr20**	21712695	21712963	269	13	-46.285482258042000	PAX1	paired box 1	4425	Intron (ENST00000398485.6/5075, intron 4 of 4)
**chr12**	57632105	57632383	279	16	-46.27103507787490	B4GALNT1	beta-1,4-N-acetyl-galactosaminyltransferase 1	264	Promoter (<=1kb)
**chr5**	124749252	124749579	328	12	-46.20750590292030	ZNF608	zinc finger protein 608	-445	Promoter (<=1kb)

**chr7**	4715184	4715414	231	14	-46.11650159107580	LOC124901581	uncharacterized LOC124901581	-10026	Intron (ENST00000328914.5/221937, intron 1 of 8)
**chr6**	101403405	101403801	397	12	-46.05202891530500	GRIK2	glutamate ionotropic receptor kainate type subunit 2	4420	Intron (ENST00000683774.1/2898, intron 6 of 16)
**chr3**	69740431	69740804	374	12	-46.04308893601610	MITF	melanocyte inducing transcription factor	949	Promoter (<=1kb)
**chr3**	138949345	138949810	466	14	-45.97950008337230	FOXL2NB	FOXL2 neighbor	1733	Promoter (1-2kb)
**chr17**	48720156	48720402	247	14	-45.91737884345370	PRAC1	PRAC1 small nuclear protein	2116	Promoter (2-3kb)
**chr2**	222320427	222320722	296	18	-45.67341938642770	CT75	cancer/testis associated transcript 75	0	Promoter (<=1kb)
**chr15**	75195592	75195981	390	14	-45.48418230057530	C15orf39	chromosome 15 open reading frame 39	0	Promoter (<=1kb)
**chr6**	3752495	3752709	215	14	-45.07509337020190	PXDC1	PX domain containing 1	-782	Promoter (<=1kb)
**chr11**	70670381	70670754	374	15	44.99385632844770	SHANK2	SH3 and multiple ankyrin repeat domains 2	-8184	Intron (ENST00000601538.6/22941, intron 15 of 25)
**chr4**	3285617	3285867	251	15	44.74877118093810	RGS12	regulator of G protein signaling 12	-7154	Distal Intergenic
**chr1**	38816131	38816345	215	15	-44.15941813158880	RRAGC	Ras related GTP binding C	31729	Distal Intergenic
**chr17**	65558334	65558685	352	17	-44.035251994797400	AXIN2	axin 2	44	Promoter (<=1kb)
**chr7**	148697578	148697893	316	11	-44.0119449071603	CUL1	cullin 1	-21	Promoter (<=1kb)
**chr4**	54108256	54108503	248	13	-43.976196323277200	GSX2	GS homeobox 2	8083	Intron (ENST00000507166.5/5156, intron 12 of 23)
**chr7**	155469640	155470019	380	10	-43.901282312632700	EN2	engrailed homeobox 2	11511	Distal Intergenic
**chr2**	131528845	131529228	384	15	-43.729120493545400	CCDC74A	coiled-coil domain containing 74A	0	Promoter (<=1kb)
**chr1**	970514	970742	229	14	-43.487711784572300	PLEKHN1	pleckstrin homology domain containing N1	-133	Promoter (<=1kb)
**chr11**	134046648	134047024	377	10	43.25885608614700	LINC02731	long intergenic non-protein coding RNA 2731	8005	Exon (ENST00000527712.2/100128239, exon 4 of 5)
**chr5**	150412630	150412917	288	13	-43.22888111974320	CD74	CD74 molecule	0	Promoter (<=1kb)
**chr20**	57398424	57398727	304	16	-43.199472762167000	RBM38-AS1	RBM38 antisense RNA 1	-5362	Intron (ENST00000356208.10/55544, intron 3 of 3)

**chr7**	155455983	155456380	398	15	-43.10637004572100	EN2	engrailed homeobox 2	-1749	Promoter (1-2kb)
**chr6**	33009572	33009931	360	11	-43.01995794441020	HLA-DOA	major histocompatibility complex, class II, DO alpha	0	Promoter (<=1kb)
**chr1**	156429404	156430082	679	12	-42.72097573111640	MIR9-1HG	MIR9-1 host gene	0	Promoter (<=1kb)
**chr8**	66114490	66114814	325	12	- 42.456480580521900	TRIM55	tripartite motif containing 55	1127	Promoter (1-2kb)
**chr6**	57173220	57173460	241	13	- 42.198554960675000	ZNF451-AS1	ZNF451 regulatory antisense RNA 1	-85	Promoter (<=1kb)
**chr20**	63126276	63126628	353	10	41.898879134726800	LOC105372719	uncharacterized LOC105372719	10058	Distal Intergenic
**chr2**	33134131	33134657	527	14	-41.75251465468200	LTBP1	latent transforming growth factor beta binding protein 1	0	Promoter (<=1kb)
**chr12**	132409811	132410019	209	13	41.51650455004390	FBRSL1	fibrosin like 1	-79532	Distal Intergenic
**chr1**	228005536	228005985	450	13	-41.34441723869550	WNT3A	Wnt family member 3A	-1013	Promoter (1-2kb)
**chr10**	43361853	43362074	222	11	-41.11334980920780	FXYD4	FXYD domain containing ion transport regulator 4	-9562	Distal Intergenic
**chr10**	3781385	3781652	268	10	40.72808379926380	KLF6	KLF transcription factor 6	399	Promoter (<=1kb)
**chr3**	42905893	42906215	323	14	- 40.654685435983300	ZNF662	zinc finger protein 662	0	Promoter (<=1kb)
**chr9**	134776555	134776836	282	10	40.47759901174610	LOC124902301	uncharacterized LOC124902301	-884	Promoter (<=1kb)
**chr7**	24574478	24574815	338	12	- 40.395901742681100	PALS2	protein associated with LIN7 2, MAGUK p55 family member	465	Promoter (<=1kb)
**chr2**	182866060	182866306	247	18	-40.37473529466570	FRZB	frizzled related protein	331	Promoter (<=1kb)
**chr2**	176172634	176172982	349	12	-40.23334617005490	HAGLR	HOXD antisense growth-associated long non-coding RNA	4586	3' UTR
**chr12**	53984144	53984381	238	14	-40.19143696739580	HOXC-AS3	HOXC cluster antisense RNA 3	377	Promoter (<=1kb)
**chr5**	135040982	135041228	247	13	-40.05618156036700	EPIST	esophagus epithelial intergenic associated transcript	-935	Promoter (<=1kb)
**chr8**	53252136	53252487	352	11	- 39.922780924988000	OPRK1	opioid receptor kappa 1	-499	Promoter (<=1kb)
**chr15**	93089916	93090179	264	12	-39.66231183693980	LOC101927025	uncharacterized LOC101927025	501	Promoter (<=1kb)

**chr2**	181954119	181954475	357	11	-39.61443316394930	PPP1R1C	protein phosphatase 1 regulatory inhibitor subunit 1C	0	Promoter (<=1kb)
**chr18**	37567800	37568520	721	12	-39.48527435918680	LOC107985118	uncharacterized LOC107985118	77	Promoter (<=1kb)
**chr5**	139646977	139647218	242	15	-39.219006687520300	CXXC5	CXXC finger protein 5	-81	Promoter (<=1kb)
**chr2**	98772426	98772883	458	10	-39.1883469807093	LINC02611	long intergenic non-protein coding RNA 2611	0	Promoter (<=1kb)
**chr2**	70905609	70905900	292	10	-38.450020345576600	ATP6V1B1	ATPase H+ transporting V1 subunit B1	4811	Intron (ENST00000234392.3/25806, intron 1 of 2)
**chr16**	54655219	54655425	207	9	-38.21869306913940	LINC02183	long intergenic non-protein coding RNA 2183	-96119	Intron (ENST00000637560.1/ENST00000637560.1, intron 1 of 4)
**chr11**	1918241	1918495	255	12	37.92867932394180	TNNT3	troponin T3, fast skeletal type	-1208	Promoter (1-2kb)
**chr4**	54095431	54095701	271	11	-37.84866329251880	GSX2	GS homeobox 2	-3822	Intron (ENST00000507166.5/5156, intron 12 of 23)
**chr1**	155974806	155976615	1810	10	-37.84080937036330	ARHGEF2	Rho/Rac guanine nucleotide exchange factor 2	1545	Promoter (1-2kb)
**chr12**	104458859	104459070	212	10	-37.69382697047390	CHST11	carbohydrate sulfotransferase 11	1858	Promoter (1-2kb)
**chr15**	69462229	69462430	202	14	37.45240308495400	DRAIC	downregulated RNA in cancer, inhibitor of cell invasion and migration	-491	Promoter (<=1kb)
**chr10**	30027933	30028257	325	10	-37.23828865202150	LOC107984218	uncharacterized LOC107984218	-26814	Exon (ENST00000375377.2/57608, exon 3 of 4)
**chr11**	115500818	115501313	496	11	-36.890434299758200	CADM1	cell adhesion molecule 1	3076	Intron (ENST00000331581.11/23705, intron 1 of 11)
**chr3**	138944372	138944624	253	10	-36.64633251537150	LINC01391	long intergenic non-protein coding RNA 1391	-352	Promoter (<=1kb)
**chr1**	46492377	46492852	476	11	-36.631978480151300	DMBX1	diencephalon/mesencephalon homeobox 1	2541	Promoter (2-3kb)
**chr9**	21970609	21970817	209	13	-36.4789530022207	CDKN2A	cyclin dependent kinase inhibitor 2A	-142	Promoter (<=1kb)
**chr17**	43543543	43544351	809	9	-36.041882149162400	ETV4	ETS variant transcription factor 4	1051	Promoter (1-2kb)

**chr20**	46199276	46199492	217	10	-35.92885501213890	CDH22	cadherin 22	21357	Exon (ENST00000537909.4/64405, exon 8 of 12)
**chr1**	6204991	6205240	250	11	-35.76298563781870	RNF207	ring finger protein 207	-235	Promoter (<=1kb)
**chr10**	132574943	132575143	201	9	-35.750870292659900	INPP5A	inositol polyphosphate-5-phosphatase A	-32783	Intron (ENST00000368594.8/3632, intron 1 of 15)
**chr6**	131250206	131250529	324	10	-35.374865425315700	AKAP7	A-kinase anchoring protein 7	0	Promoter (<=1kb)
**chr12**	76030016	76030426	411	9	-35.04384258230700	PHLDA1	pleckstrin homology like domain family A member 1	1178	Promoter (1-2kb)
**chr1**	156432736	156433150	415	12	-35.02425743138430	MIR9-1HG	MIR9-1 host gene	-2035	Promoter (2-3kb)
**chr14**	36515072	36515372	301	9	-34.94264121735530	SFTA3	surfactant associated 3	-1243	Promoter (1-2kb)
**chr2**	221569502	221569787	286	12	-34.81335368917230	EPHA4	EPH receptor A4	-21	Promoter (<=1kb)
**chr4**	54098371	54099043	673	10	-34.56638222731390	GSX2	GS homeobox 2	-480	Promoter (<=1kb)
**chr10**	49649407	49649618	212	12	-34.497500502482400	CHAT	choline O-acetyltransferase	-2118	Promoter (2-3kb)
**chr10**	101218858	101219131	274	14	-34.495377475314	LBX1	ladybird homeobox 1	10332	Distal Intergenic
**chr16**	54935637	54936012	376	11	-34.35040644742100	LOC124903692	uncharacterized LOC124903692	2659	Promoter (2-3kb)
**chr1**	46448662	46448886	225	10	-34.34824429970250	LINC01398	long intergenic non-protein coding RNA 1398	818	Promoter (<=1kb)
**chr12**	86264431	86264681	251	13	-34.305436235133800	MGAT4C	MGAT4 family member C	-8017	Intron (ENST00000548651.6/25834, intron 4 of 7)
**chr4**	173507763	173508048	286	12	-34.235917382245600	LOC101928409	uncharacterized LOC101928409	-24	Promoter (<=1kb)
**chr14**	52313414	52313711	298	10	-33.88399123063670	PTGER2	prostaglandin E receptor 2	-594	Promoter (<=1kb)
**chr11**	69444894	69445142	249	13	-33.50792459532790	LINC02953	long intergenic non-protein coding RNA 2953	19216	Distal Intergenic
**chr6**	73654772	73655087	316	7	33.05859198667050	SLC17A5	solute carrier family 17 member 5	-780	Promoter (<=1kb)
**chr2**	70898391	70899033	643	10	-32.82201344714250	VAX2	ventral anterior homeobox 2	-1543	Promoter (1-2kb)
**chr11**	45145672	45145942	271	10	-32.40552510195800	PRDM11	PR/SET domain 11	-697	Promoter (<=1kb)
**chr13**	27976401	27976803	403	9	-32.15361120622010	CDX2	caudal type homeobox 2	-7086	Distal Intergenic
**chr16**	23837118	23837381	264	11	-31.99207240589200	PRKCB	protein kinase C beta	0	Promoter (<=1kb)

**chr8**	28353337	28353543	207	11	31.899476226699600	ZNF395	zinc finger protein 395	-83	Promoter (<=1kb)
**chr3**	129305591	129305955	365	11	-31.85567410783280	H1-10-AS1	H1-10 antisense RNA 1	-9437	3' UTR
**chr2**	172092714	172093044	331	8	-31.585449294914700	DLX1	distal-less homeobox 1	5347	Exon (ENST00000686675.1/ENST00000686675.1, exon 1 of 1)
**chr4**	173523931	173524198	268	9	-31.536777030424500	HAND2-AS1	HAND2 antisense RNA 1	-3072	Distal Intergenic
**chr22**	37675471	37675678	208	11	-31.510642339560400	LGALS1	galectin 1	0	Promoter (<=1kb)
**chr5**	67234669	67234880	212	7	-31.483987955648800	CD180	CD180 molecule	-37870	Distal Intergenic
**chr11**	32432820	32433112	293	9	-31.36165764984710	WT1	WT1 transcription factor	1056	Promoter (1-2kb)
**chr11**	33868565	33868774	210	9	-31.322371971947400	LMO2	LIM domain only 2	762	Promoter (<=1kb)
**chr14**	85535189	85535611	423	9	-31.127692718387400	FLRT2	fibronectin leucine rich transmembrane protein 2	1228	Promoter (1-2kb)
**chr21**	35049560	35049815	256	7	-31.042145879620900	RUNX1	RUNX family transcription factor 1	-216	Promoter (<=1kb)
**chr4**	88737380	88737744	365	8	-30.87831010140800	FAM13A	family with sequence similarity 13 member A	0	Promoter (<=1kb)
**chr4**	124713341	124713637	297	8	-30.81881917095370	ANKRD50	ankyrin repeat domain containing 50	-609	Promoter (<=1kb)
**chr1**	24112405	24112668	264	12	-30.501529974221300	MYOM3	myomesin 3	-270	Promoter (<=1kb)
**chr21**	29080408	29080691	284	7	-30.44144337777560	MAP3K7CL	MAP3K7 C-terminal like	0	Promoter (<=1kb)
**chr2**	86037113	86037430	318	8	-29.997559583408800	POLR1A	RNA polymerase I subunit A	-43	Promoter (<=1kb)
**chr5**	160133516	160133722	207	9	-29.881071882259000	PWWP2A	PWWP domain containing 2A	-14066	Distal Intergenic
**chr3**	106899075	106899290	216	8	29.850339259828500	LOC105374029	uncharacterized LOC105374029	-207755	Exon (ENST00000460269.1/ENST00000460269.1, exon 1 of 1)
**chr2**	172090987	172091241	255	9	-29.499831915983000	DLX1	distal-less homeobox 1	3620	Distal Intergenic

**chr2**	176125771	176126019	249	7	-29.45940477297670	HOXD9	homeobox D9	3052	Intron (ENST00000440016.6/100506783, intron 2 of 3)
**chr14**	100618170	100618446	277	8	-29.34305526897960	BEGAIN	brain enriched guanylate kinase associated	-30753	Distal Intergenic
**chr11**	17015097	17015302	206	9	-29.319921953269900	PLEKHA7	pleckstrin homology domain containing A7	-682	Promoter (<=1kb)
**chr11**	72245319	72245609	291	10	-28.974961286569300	PHOX2A	paired like homeobox 2A	55	Promoter (<=1kb)
**chr10**	73591318	73592130	813	7	-28.805575211202300	USP54	ubiquitin specific peptidase 54	0	Promoter (<=1kb)
**chr7**	20777001	20777254	254	9	-28.132298270823100	SP8	Sp8 transcription factor	9632	3' UTR
**chr12**	49337815	49338102	288	9	-28.11822189754960	C1QL4	complement C1q like 4	-627	Promoter (<=1kb)
**chr10**	101130868	101131161	294	9	-27.4050335736335	TLX1NB	TLX1 neighbor	0	Promoter (<=1kb)
**chr1**	91804849	91809624	4776	9	27.38592292372430	TGFBR3	transforming growth factor beta receptor 3	-38417	Intron (ENST00000533089.5/7049, intron 1 of 19)
**chr14**	24315581	24315799	219	8	27.378657490929800	LTB4R	leukotriene B4 receptor	881	Promoter (<=1kb)
**chr10**	61000854	61001107	254	8	-27.230518467986900	LINC00845	long intergenic non-protein coding RNA 845	-10	Promoter (<=1kb)
**chr5**	73443675	73443912	238	7	-27.202058790615600	FOXD1	forkhead box D1	4865	Distal Intergenic
**chr9**	128176370	128176574	205	6	27.137775157381500	CIZ1	CDKN1A interacting zinc finger protein 1	1034	Promoter (1-2kb)
**chr20**	63346374	63346629	256	9	27.106540049484100	CHRNA4	cholinergic receptor nicotinic alpha 4 subunit	7937	3' UTR
**chr19**	56156675	56156925	251	8	27.042322555342600	ZNF444	zinc finger protein 444	-384	Promoter (<=1kb)
**chr14**	36588942	36589152	211	7	-26.694124889469200	NKX2-8	NK2 homeobox 8	-6328	Intron (ENST00000634305.1/ENST00000634305.1, intron 3 of 3)
**chr2**	27096251	27096506	256	8	-26.664370299805600	KHK	ketohexokinase	1586	Promoter (1-2kb)
**chr4**	173535128	173535516	389	9	-26.491756264113200	HAND2-AS1	HAND2 antisense RNA 1	3396	Intron (ENST00000512099.5/79804, intron 2 of 3)
**chr6**	114341294	114341512	219	7	-26.44116032676410	HS3ST5	heparan sulfate-glucosamine 3-sulfotransferase 5	1511	Promoter (1-2kb)

**chr8**	22554078	22554300	223	9	-26.267737684824700	SORBS3	sorbin and SH3 domain containing 3	0	Promoter (<=1kb)
**chr15**	92097609	92097866	258	10	-26.258851287897700	SLCO3A1	solute carrier organic anion transporter family member 3A1	-64	Promoter (<=1kb)
**chr15**	96370128	96370357	230	7	-26.120191205805400	LOC101927263	uncharacterized LOC101927263	-2176	Promoter (2-3kb)
**chr5**	79512076	79512307	232	9	-26.115597603636300	HOMER1	homer scaffold protein 1	487	Promoter (<=1kb)
**chr16**	10877178	10877393	216	7	-26.02570041286080	CIITA	class II major histocompatibility complex transactivator	0	Promoter (<=1kb)
**chr9**	21988688	21989200	513	8	-25.678670770146200	CDKN2B-AS1	CDKN2B antisense RNA 1	-4939	Intron (ENST00000404796.3/4507, intron 4 of 4)
**chr10**	132910740	132910979	240	10	25.641947725833000	CFAP46	cilia and flagella associated protein 46	-644	Promoter (<=1kb)
**chr20**	25497469	25497700	232	8	25.544284603533900	NINL	ninein like	554	Promoter (<=1kb)
**chr20**	21707434	21707745	312	7	-25.212879455850600	PAX1	paired box 1	-525	Promoter (<=1kb)
**chr17**	50944111	50944336	226	7	-25.20098448245910	SPAG9	sperm associated antigen 9	31024	Exon (ENST00000506394.1/ENST00000506394.1, exon 1 of 2)
**chr13**	106532129	106532463	335	9	-25.18959355638140	EFNB2	ephrin B2	3199	Intron (ENST00000646441.1/1948, intron 1 of 4)
**chr2**	108377855	108378166	312	8	-25.06639516896960	SULT1C4	sulfotransferase family 1C member 4	0	Promoter (<=1kb)
**chr5**	137500652	137500900	249	9	25.011355361779000	SPOCK1	SPARC (osteonectin), cwcv and kazal like domains proteoglycan 1	-1326	Promoter (1-2kb)
**chr12**	7369417	7369636	220	8	24.79873857239720	CD163L1	CD163 molecule like 1	0	Promoter (<=1kb)
**chr1**	169107455	169107806	352	7	-24.79582352289900	ATP1B1	ATPase Na+/K+ transporting subunit beta 1	0	Promoter (<=1kb)
**chr1**	164589013	164589232	220	7	-24.58140012302040	PBX1	PBX homeobox 1	-1118	Promoter (1-2kb)
**chr19**	608881	609173	293	9	24.557358411317700	POLRMT	RNA polymerase mitochondrial	8824	Intron (ENST00000251287.3/610, intron 4 of 7)
**chr5**	88668068	88668301	234	8	-24.554672709831100	MIR9-2	microRNA 9-2	-1129	Promoter (1-2kb)

**chr9**	35757405	35757643	239	8	-24.35457017309440	MSMP	microseminoprotein, prostate associated	-792	Promoter (<=1kb)
**chr19**	58554133	58554338	206	9	24.30246011782280	CHMP2A	charged multivesicular body protein 2A	300	Promoter (<=1kb)
**chr9**	32604003	32604337	335	6	24.10626092481690	NDUFB6	NADH:ubiquinone oxidoreductase subunit B6	-30819	Distal Intergenic
**chr1**	54547608	54547844	237	6	-24.055389459028300	ACOT11	acyl-CoA thioesterase 11	-376	Promoter (<=1kb)
**chr1**	218674480	218674732	253	6	-24.035576453116300	LOC124904666	small nucleolar RNA U3	-132581	Distal Intergenic
**chr5**	142769405	142769661	257	8	-24.033284988482700	ARHGAP26	Rho GTPase activating protein 26	-716	Promoter (<=1kb)
**chr12**	114690227	114690761	535	7	-23.743728363792900	TBX3	T-box transcription factor 3	-6052	Intron (ENST00000660721.1/ENST00000660721.1, intron 1 of 1)
**chr6**	30940802	30941010	209	7	23.738663057716800	MUCL3	mucin like 3	0	Promoter (<=1kb)
**chr5**	77087959	77088232	274	9	-23.6450681041561	ZBED3-AS1	ZBED3 antisense RNA 1	0	Promoter (<=1kb)
**chr18**	54959012	54959245	234	10	-23.642445366542900	CCDC68	coiled-coil domain containing 68	216	Promoter (<=1kb)
**chr8**	76672786	76672987	202	7	-23.512198992428100	ZFHX4-AS1	ZFHX4 antisense RNA 1	0	Promoter (<=1kb)
**chr3**	107598907	107599259	353	5	-23.229013701449100	BBX	BBX high mobility group box domain containing	-54	Promoter (<=1kb)
**chr8**	85178334	85178542	209	9	-22.212649629365900	E2F5	E2F transcription factor 5	722	Promoter (<=1kb)
**chr11**	67404899	67405147	249	7	-22.113197210901500	TBC1D10C	TBC1 domain family member 10C	0	Promoter (<=1kb)
**chr8**	70217213	70217515	303	5	-22.041020514095100	NCOA2	nuclear receptor coactivator 2	27860	Intron (ENST00000452400.7/10499, intron 2 of 22)
**chr4**	41256447	41256647	201	6	-21.770708497566100	UCHL1	ubiquitin C-terminal hydrolase L1	34	Promoter (<=1kb)
**chr1**	156437584	156437826	243	6	-21.220801900914400	MIR9-1HG	MIR9-1 host gene	-6883	Intron (ENST00000673141.1/10485, intron 2 of 8)
**chr5**	73418883	73419122	240	6	-21.01662900376610	FOXD1	forkhead box D1	29655	Distal Intergenic

**chr2**	84907137	84907579	443	8	-20.799568701246800	LOC105374836	uncharacterized LOC105374836	-129	Promoter (<=1kb)
**chr4**	173524212	173524449	238	7	-20.742375754226200	HAND2-AS1	HAND2 antisense RNA 1	-2821	Promoter (2-3kb)
**chr2**	176156516	176156749	234	7	-20.4861957303886	HOXD3	homeobox D3	-558	Promoter (<=1kb)
**chr12**	132305442	132305698	257	7	20.222156284543700	GALNT9	polypeptide N-acetylgalactosaminyltransferase 9	-18971	Intron (ENST00000328957.13/50614, intron 1 of 10)
**chr2**	241965952	241966183	232	5	-20.083504836747900	LINC01238	long intergenic non-protein coding RNA 1238	-4500	Exon (ENST00000430555.5/101927289, exon 4 of 4)
**chr6**	16064692	16064942	251	5	-19.940350087753100	MYLIP	myosin regulatory light chain interacting protein	-64144	Distal Intergenic
**chr16**	89330511	89330755	245	5	-19.878234579009100	ANKRD11	ankyrin repeat domain containing 11	-1525	Promoter (1-2kb)
**chr6**	30452699	30452917	219	7	-19.7726355986102	LOC105375012	uncharacterized LOC105375012	28717	Intron (ENST00000658719.1/ENST00000658719.1, intron 1 of 1)
**chr15**	60590324	60590737	414	5	19.710982144654400	RORA	RAR related orphan receptor A	1780	Promoter (1-2kb)
**chr2**	176102893	176103422	530	6	-19.685665184735000	HOXD11	homeobox D11	-794	Promoter (<=1kb)
**chr19**	54664836	54665074	239	8	19.473314772209100	LILRB4	leukocyte immunoglobulin like receptor B4	-724	Promoter (<=1kb)
**chr3**	196639056	196639295	240	6	-19.28225548650330	NRROS	negative regulator of reactive oxygen species	-399	Promoter (<=1kb)
**chr19**	35139202	35139430	229	5	-18.84532153254690	FXYD1	FXYD domain containing ion transport regulator 1	-11	Promoter (<=1kb)
**chr9**	135274204	135274420	217	6	18.460922499450700	LOC107987138	uncharacterized LOC107987138	-21496	Distal Intergenic
**chr22**	17191203	17191414	212	6	18.37420719278860	ADA2	adenosine deaminase 2	396	Promoter (<=1kb)
**chr1**	156430354	156430695	342	5	-18.279330823000700	MIR9-1HG	MIR9-1 host gene	6	Promoter (<=1kb)
**chr17**	57956395	57956615	221	6	-17.93410905965630	CUEDC1	CUE domain containing 1	-983	Promoter (<=1kb)
**chr20**	47348327	47348706	380	4	-17.863554662564900	ZMYND8	zinc finger MYND-type containing 8	-255	Promoter (<=1kb)

**chr10**	31137502	31137768	267	5	-17.8395229284587	LOC105376483	uncharacterized LOC105376483	3638	Intron (ENST00000658872.1/105376481, intron 2 of 2)
**chr16**	88432467	88432735	269	5	17.476549231386500	ZFPM1	zinc finger protein, FOG family member 1	-20545	Exon (ENST00000565624.3/84627, exon 3 of 3)
**chr21**	41186728	41187092	365	5	16.42144093512190	PLAC4	placenta enriched 4	-1489	Promoter (1-2kb)
**chr22**	19167390	19167592	203	4	16.401695775562300	LINC01311	long intergenic non-protein coding RNA 1311	-3803	Distal Intergenic
**chr5**	180611733	180611979	247	4	16.046345568215300	FLT4	fms related receptor tyrosine kinase 4	-17	Promoter (<=1kb)
**chr7**	94427040	94427266	227	4	15.941292124640600	COL1A2	collagen type I alpha 2 chain	-46	Promoter (<=1kb)
**chr10**	88852471	88852679	209	4	15.899658935596300	ANKRD22	ankyrin repeat domain 22	-627	Promoter (<=1kb)
**chr17**	76561412	76561621	210	5	15.600168798077600	SNORD1A	small nucleolar RNA, C/D box 1A	-13	Promoter (<=1kb)
**chr10**	73911918	73912159	242	4	14.797667096792100	PLAU	plasminogen activator, urokinase	771	Promoter (<=1kb)
**chr1**	29709371	29709613	243	4	-14.490568029830200	LINC01648	long intergenic non-protein coding RNA 1648	327999	Exon (ENST00000422638.1/ENST00000422638.1, exon 1 of 2)
**chr20**	61255065	61255291	227	5	14.316449453622000	CDH4	cadherin 4	2804	Promoter (2-3kb)
**chr18**	78393361	78393575	215	4	14.23254230742920	LINC01029	long intergenic non-protein coding RNA 1029	-399630	Distal Intergenic
**chr4**	1798027	1798396	370	5	13.352593234077300	FGFR3	fibroblast growth factor receptor 3	-3066	Intron (ENST00000340107.9/2261, intron 2 of 17)
**chr8**	115666355	115666579	225	4	-13.141727300437000	TRPS1	transcriptional repressor GATA binding 1	1402	Promoter (1-2kb)
**chr3**	141076735	141076981	247	4	-12.528457075439500	SPSB4	splA/ryanodine receptor domain and SOCS box containing 4	0	Promoter (<=1kb)
**chr1**	156662063	156662320	258	4	-12.455273654790300	BCAN-AS2	BCAN antisense RNA 2	-639	Promoter (<=1kb)
**chr1**	63746310	63746510	201	4	-12.208140243566500	ROR1	receptor tyrosine kinase like orphan receptor 1	-27507	Distal Intergenic

Supplemental table 2: Time-series differentially methylated regions (DMRs) associated with skin-relevant genes.

chr	start	end	length	nCG	areaStat	SYMBOL	GENENAME	distanceToTSS	annotation	function_summary
**chr17**	72122660	72124417	1758	107	-425.5025007	SOX9	SRY-box transcription factor 9	1640	Promoter (1-2kb)	TF regulating epidermal stem cells, hair follicle stem cell maintenance and keratinocyte biology
**chr22**	45971323	45972630	1308	139	-423.2846592	WNT7B	Wnt family member 7B	85	Promoter (<=1kb)	Wnt ligand; regulator of hair follicle stem cells and epidermal Wnt signalling
**chr1**	243482609	243483585	977	72	-253.9593955	SDCCAG8	SHH signaling and ciliogenesis regulator SDCCAG8	-4230	Intron (ENST00000366541.8/10806, intron 16 of 17)	Ciliogenesis/SHH pathway regulator (needed for proper cilia function; cilia important in skin signalling)
**chr17**	17722541	17723204	664	55	-194.9415388	RAI1	retinoic acid induced 1	40046	Intron (ENST00000353383.6/10743, intron 1 of 5)	Dosage-sensitive regulator (RAI1) — linked to retinoic-acid pathways and has indirect effects on lipid/skin gene expression
**chr9**	88990630	88991299	670	45	-187.8468242	S1PR3	sphingosine-1-phosphate receptor 3	0	Promoter (<=1kb)	S1P receptor subtype implicated in keratinocyte survival, inflammation and wound healing
**chr16**	68642548	68643122	575	46	-180.0365005	CDH3	cadherin 3	-1871	Promoter (1-2kb)	P-cadherin (CDH3) — adherens junction protein important for hair follicle and epidermal morphogenesis
**chr12**	43550829	43551351	523	37	-164.290376	ADAMTS20	ADAM metallopeptidase with thrombospondin type 1 motif 20	570	Promoter (<=1kb)	Secreted metalloprotease required for melanoblast survival and melanocyte development
**chr2**	30230560	30231133	574	38	-139.1432399	LBH	LBH regulator of WNT signaling pathway	-401	Promoter (<=1kb)	Wnt-responsive transcriptional regulator involved in epidermal/hair follicle signalling
**chr4**	153792726	153793262	537	35	-134.0056695	SFRP2	secreted frizzled related protein 2	-3643	Distal Intergenic	Secreted Wnt modulator involved in fibroblast signalling, pigmentation and fibrosis (modulates Wnt responses)
**chr2**	120741751	120742134	384	42	-126.2573763	GLI2	GLI family zinc finger 2	5883	Intron (ENST00000482119.6/2736, intron 1 of 3)	Hedgehog pathway TF required for hair follicle development and epidermal proliferation
**chr12**	54417971	54418824	854	26	-103.0577642	ITGA5	integrin subunit alpha 5	303	Promoter (<=1kb)	Integrin α5 (α5β1) — mediates keratinocyte/fibroblast adhesion and migration during repair

**chr4**	1802514	1802998	485	31	101.9738364	FGFR3	fibroblast growth factor receptor 3	602	Promoter (<=1kb)	FGF receptor with roles in epidermal homeostasis; activating FGFR3 mutations affect epidermis
**chr6**	36285008	36285602	595	28	-101.188206	PNPLA1	patatin like phospholipase domain containing 1	14548	Intron (ENST00000312917.9/285848, intron 1 of 7)	Transacylase required for acylceramide biosynthesis and epidermal lipid barrier (ichthyosis gene)
**chr2**	237486290	237486699	410	33	-98.57563	MLPH	melanophilin	0	Promoter (<=1kb)	Melanophilin — melanosome transport adaptor critical for melanosome distribution/pigmentation
**chr3**	137032600	137032967	368	22	-97.26726564	IL20RB-AS1	IL20RB antisense RNA 1	-50404	Distal Intergenic	IL-20 receptor pathway (lncRNA here is IL20RB-associated) — IL-20/IL20R signalling is skin-relevant (psoriasis, keratinocyte responses)
**chr6**	168442248	168442599	352	25	-95.85837385	SMOC2	SPARC related modular calcium binding 2	1064	Promoter (1-2kb)	Matricellular protein (SPARC family) implicated in wound healing, ECM interactions and angiogenesis
**chr3**	139538768	139539167	400	23	-81.02975505	RBP1	retinol binding protein 1	469	Promoter (<=1kb)	Cellular retinol-binding protein involved in retinoid handling in skin / epidermal differentiation
**chr8**	41310141	41310817	677	21	-79.5514491	SFRP1	secreted frizzled related protein 1	-668	Promoter (<=1kb)	Secreted Wnt antagonist; modulates wound healing and keratinocyte apoptosis (Wnt pathway regulator)
**chr14**	53952754	53953148	395	24	-78.08065833	BMP4	bone morphogenetic protein 4	281	Promoter (<=1kb)	BMP family member; regulates melanocyte differentiation and hair follicle/epidermal differentiation
**chr4**	54656941	54657407	467	22	-71.1277294	KIT	KIT proto-oncogene, receptor tyrosine kinase	0	Promoter (<=1kb)	Receptor tyrosine kinase (c-KIT) essential for melanocyte survival, pigment production and hair pigmentation
**chr6**	168384490	168384952	463	20	-63.64636134	SMOC2	SPARC related modular calcium binding 2	-56199	Distal Intergenic	Matricellular protein (SPARC family) implicated in wound healing, ECM interactions and angiogenesis
**chr9**	107491257	107491560	304	21	-63.44714424	KLF4	KLF transcription factor 4	-775	Promoter (<=1kb)	TF required for keratinocyte differentiation and skin barrier formation
**chr4**	1798705	1799514	810	20	53.72165947	FGFR3	fibroblast growth factor receptor 3	-1948	Promoter (1-2kb)	FGF receptor with roles in epidermal homeostasis; activating FGFR3 mutations affect epidermis

**chr16**	55480344	55480597	254	17	-51.90190226	MMP2	matrix metallopeptidase 2	0	Promoter (<=1kb)	Matrix metalloproteinase involved in ECM remodelling and wound healing
**chr6**	75202526	75202995	470	17	-51.5884855	COL12A1	collagen type XII alpha 1 chain	0	Promoter (<=1kb)	Fibril-associated collagen (XII) that regulates dermal matrix organization and wound repair
**chr3**	69740431	69740804	374	12	-46.04308894	MITF	melanocyte inducing transcription factor	949	Promoter (<=1kb)	Master TF for melanocyte development, pigmentation and melanocyte lineage identity
**chr1**	228005536	228005985	450	13	-41.34441724	WNT3A	Wnt family member 3A	-1013	Promoter (1-2kb)	Canonical Wnt ligand; influences keratinocyte proliferation and wound healing
**chr10**	3781385	3781652	268	10	40.7280838	KLF6	KLF transcription factor 6	399	Promoter (<=1kb)	Zinc-finger TF involved in injury response and repair-related gene expression
**chr21**	35049560	35049815	256	7	-31.04214588	RUNX1	RUNX family transcription factor 1	-216	Promoter (<=1kb)	Transcription factor that promotes hair follicle stem cell proliferation and activation
**chr1**	91804849	91809624	4776	9	27.38592292	TGFBR3	transforming growth factor beta receptor 3	-38417	Intron (ENST00000533089.5/7049, intron 1 of 19)	TGF-β co-receptor (betaglycan) — modulates TGF-β signalling in fibrosis / wound healing
**chr5**	137500652	137500900	249	9	25.01135536	SPOCK1	SPARC (osteonectin), cwcv and kazal like domains proteoglycan 1	-1326	Promoter (1-2kb)	Proteoglycan (Testican) that modulates ECM and MMP activity (ECM regulator)
**chr7**	94427040	94427266	227	4	15.94129212	COL1A2	collagen type I alpha 2 chain	-46	Promoter (<=1kb)	Fibrillar collagen type I alpha-2; major dermal ECM component (skin strength/aging)
**chr10**	73911918	73912159	242	4	14.7976671	PLAU	plasminogen activator, urokinase	771	Promoter (<=1kb)	Urokinase plasminogen activator (uPA) — ECM proteolysis, keratinocyte migration and wound remodeling
**chr20**	61255065	61255291	227	5	14.31644945	CDH4	cadherin 4	2804	Promoter (2-3kb)	R-cadherin (CDH4) — cadherin family member with roles in epithelial adhesion/motility
**chr4**	1798027	1798396	370	5	13.35259323	FGFR3	fibroblast growth factor receptor 3	-3066	Intron (ENST00000340107.9/2261, intron 2 of 17)	FGF receptor with roles in epidermal homeostasis; activating FGFR3 mutations affect epidermis

Supplemental Table 3: Fisher's exact test results for enrichment of age-related loci in reference databases

Age Direction	NAFL Direction	Observed Overlap	Expected Overlap	Fold Enrichment	Odds Ratio	OR 95% CI	Fisher p	Permutation p	Permutation z
**hyper**	hyper	11980	19362.8	0.62	0.57	[0.56, 0.58]	<2e-16	< 1e-4	-58.67
**hypo**	hyper	56521	35103.5	1.61	2.21	[2.19, 2.24]	<2e-16	< 1e-4	139.52
**hypo**	hypo	15039	43863.6	0.34	0.26	[0.26, 0.26]	<2e-16	< 1e-4	-167.07
**hyper**	hypo	84675	24194.9	3.5	7.64	[7.56, 7.72]	<2e-16	< 1e-4	437.9
